# Cation-Induced Interphasial Viscosity Variations on Gold Electrocatalysts in Nanoconfined Aqueous Electrolytes

**DOI:** 10.1021/jacs.6c06065

**Published:** 2026-06-12

**Authors:** Martin Munz, Shane Carlson, Leon Jacobse, Roland R. Netz, Beatriz Roldan-Cuenya, Christopher S. Kley

**Affiliations:** † Helmholtz Young Investigator Group Nanoscale Operando CO2 Photo-Electrocatalysis, Helmholtz-Zentrum Berlin für Materialien und Energie GmbH, 14109 Berlin, Germany; ‡ Department of Interface Science, Fritz Haber Institute of the Max Planck Society, 14195 Berlin, Germany; § Fachbereich Physik, 9166 Freie Universität Berlin, Arnimallee 14, 14195 Berlin, Germany

## Abstract

The structure and viscosity of interfacial hydration layers critically govern charge-transfer, kinetics, and molecular diffusivity in electrocatalysis and energy conversion. Yet, despite their relevance particularly in confined catalysis, the viscosity variations across interfacial zones, of a finite width (interphases), remain largely unexplored. Here, we combine localized friction force analysis and molecular dynamics simulations, to reveal how alkali metal cations influence the hydrogen-bond network of gold–electrolyte interfaces under nanoconfinement. For potassium cations (K^+^) in aqueous perchlorate electrolyte, friction decreases linearly with increasing electrolyte concentration, evidencing a lubricating effect. Simulated density and viscosity profiles for chloride electrolytes show that chaotropic K^+^ ions weaken the hydrogen-bond network, similarly to cesium cations (Cs^+^). The interphasial layer exhibits a zone of reduced density and viscosity, followed by an adjacent zone where the viscosity clearly rises above the bulk level. These molecular-level insights are broadly relevant to understanding and quantitatively describing interphasial molecular mobility in catalysis and electrochemical sensing.

## Graphical abstract

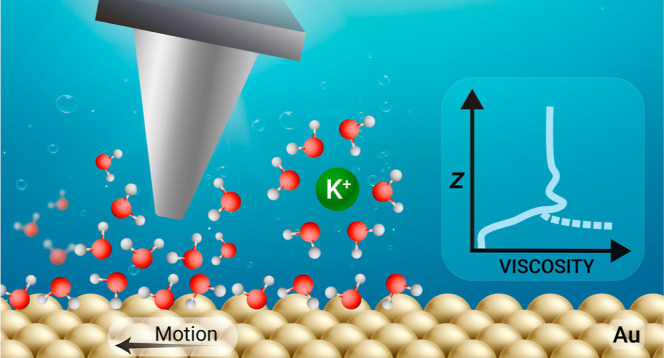

## 1 Introduction

The selectivity and kinetics of electro- and biocatalytic reactions are strongly governed by interfacial hydration layers at the catalyst–electrolyte interfaces. Proton and electron transfer processes are highly sensitive to the structure of the hydrogen-bonding network within the electrical double layer, which depends on electrolyte composition, cation/anion identities, or surface-adsorbed species.^1–3^ In addition, this interfacial molecular ordering influences the local electrolyte viscosity, which controls the diffusivity of reactants and intermediates via suitably generalized Stokes–Einstein relations.^4–6^ As a result, reaction rates are modulated by the viscosity profile: increased local viscosity reduces molecular diffusivity,^7–9^ thus limiting reactant transport to the catalyst–electrolyte interface^10^ and exchange of intermediates with the bulk solution.^11,12^ Viscosity effects are particularly pronounced under nanoconfinement, such as in enzyme active sites^13,14^ or zeolitic frameworks.^15,16^ Confinement can occur in pore-like or slit-like geometries and is critical in applications ranging from electrocatalysis to desalination membranes and nanofluidics.^17^ Within ∼1 nm of a solid polar surface, the viscosity of aqueous solutions was found to increase significantly,^4,18–23^ forming an interphasial (rather than purely interfacial) region whose properties differ markedly from the bulk. Despite its central role in microkinetic processes, the spatial variation of local electrolyte viscosity remains largely unresolved.

Ions in aqueous electrolytes can reorganize hydration layers within the inner and outer Helmholtz plane of the electric double layer.^3,24–28^ Such weakening or strengthening of the hydrogen bond (HB) network has a direct impact on, for instance, the CO_2_ electroreduction reaction (CO_2_RR), as demonstrated on gold^29^ and copper^30,31^ catalysts, where the structure of interphasial hydration layers strongly influences reaction kinetics and selectivity. In acidic electrolytes, increased K^+^ concentrations hinder proton transport to the interface, suppressing the hydrogen evolution reaction (HER).^32^ Increasing interfacial cation density can enhance CO_2_RR while suppressing competing HER pathways.^33,34^ Beyond these specific ion effects, the ordering of interfacial water also influences electron-transfer kinetics, thus further modulating electrochemical reaction rates.^35–37^ Although the influence of interfacial hydration layers on electron transfer has been examined experimentally^38,39^ and theoretically,^40,41^ disentangling their effect on local viscosity and associated molecular mobility remains a persistent challenge.

Addressing these open questions requires experimental techniques capable of resolving interphasial viscosity and its modulation by anions or cations. While vibrational spectroscopy has demonstrated the critical role of hydration-layer structure in electrochemical processes,^42–45^ interfacial properties such as viscosity and conductivity remain poorly understood.^46^ Nuclear magnetic resonance can probe molecular diffusivity^47,48^ but lacks the spatial resolution needed to capture nanoscale interfacial variations. By contrast, friction force measurements in liquid environment provide a direct probe of interphasial chemical-mechanical properties, as the measured friction depends on the properties of the liquid confined between the tip and the sample surface.^49,50^ Friction force imaging, optionally combined with in situ conductive AFM, can be particularly sensitive to the presence of ions.^51^

In this work, we investigate how alkali metal cations modulate the interphasial viscosity of polycrystalline gold in aqueous electrolytes (KClO_4_), focusing on the mildly structure-breaking (chaotropic) K^+^, a prototypical cation in CO_2_RR.^35,36,52^ Using in situ AFM, we quantify friction variations with electrolyte concentration, providing a direct probe of interfacial molecular mobility. To move beyond a qualitative explanation of the observed boundary-lubrication effects from the chaotropic nature of K^+^ and ClO_4_^–^ ions, we employ molecular dynamics (MD) simulations for revealing nanoscale viscosity and density profiles near the surface. Showing reduced viscosity in the first hydration layer followed by a peak in the adjacent zone, key features of these profiles scale with ion radius and correlate with the Jones–Dole *B*-coefficient, thus linking molecular-scale hydration structuring to the chao- and kosmotropic character of cations.

## 2 Results and Discussion

For AFM friction force measurements, the rectangular microcantilever and sample surface were fully immersed into the electrolyte, while a reciprocating lateral motion (perpendicular to the cantilever’s long axis) of the sample was generated (Figure 1a,c). Gold was chosen as the electrode material for its high chemical stability and widespread use in electrocatalysis, including CO_2_RR,^51,53,54^ and chemical sensing.^15,55^ To probe ion effects on interphasial viscosity, we scanned polycrystalline gold films with predominant (111) orientation (Figure S1), in deionized water and aqueous KClO_4_ electrolytes of varying concentrations. The gold surface height image (Figures 1b and S2a) exhibited an RMS roughness of ∼ 0.9 nm and grain sizes in the range of 3.4–40.4 nm (Figure S2c,e). Friction loops of 600 nm scan width (Figures 1c and S3) were recorded as a function of applied load and electrolyte concentration, focusing on the low-concentration regime (≤25 mM, Figures 1 and 2), where the confined hydrogen-bond (HB) network is not saturated and ion–ion friction remains negligible.^56^ All measurements were conducted at open-circuit potential (i.e., just slightly above the approximate potential of zero charge *E*_pzc_ ∼ −99 mV vs Ag, corresponding to ∼ −49 mV vs Ag/AgCl (Figure S4)), to eliminate the need for counter or reference electrodes and related risk of ion release. During contact-mode scanning,^57,58^ liquid molecules are confined within the tip/electrolyte/sample nanogap (Figure 1a), representing a slit-pore geometry. In this configuration, the mechanical resistance experienced by the AFM tip decreases if the HB network of the interfacial hydration layer is weakened by ions.

**Figure 1:**
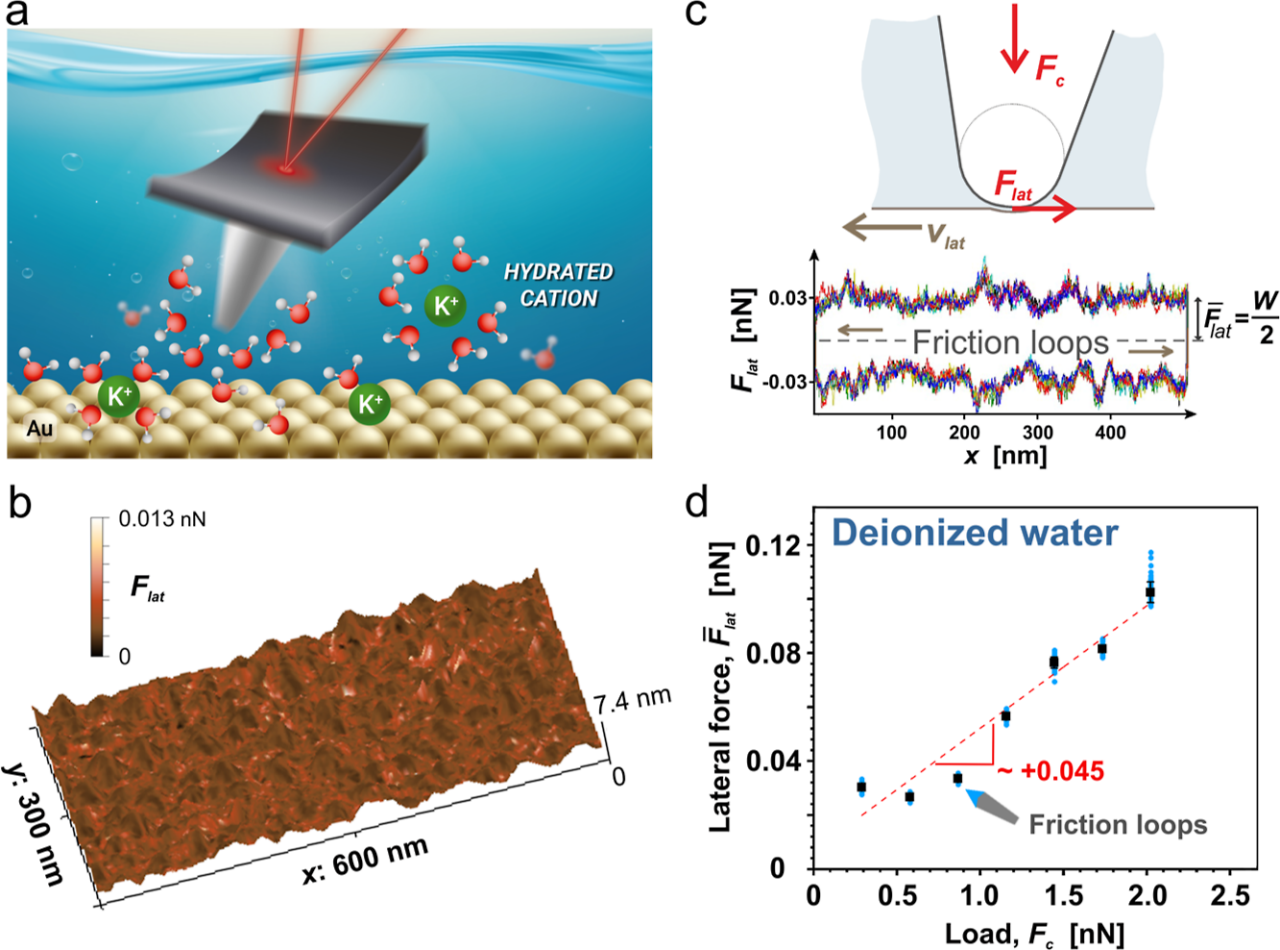
In situ AFM friction force measurements. (a) Schematic of the tip/electrolyte/gold nanogap, mimicking nanoconfinement with interfacial water and hydrated K^+^. (b) 3D-rendered height image of the gold catalyst surface, overlaid with the corresponding friction force image. (c) Schematic of a friction force measurement when the sample scanning is from right to left (at scan speed *v*_lat_, forces *F*_c_ and *F*_lat_) and individual friction loops (at *F*_c_ ∼ 0.87 nN, half-width ${\mathrm{F̅}}_{lat}=W/2$ of ∼0.034 nN). (d) Plot of the mean lateral force vs normal force in deionized water, including individual values (dots, *light blue*) and mean values (squares, *black*); linear regression curve (*dashed* line, *red*) with a slope $\Delta {\mathrm{F̅}}_{lat}/\Delta F_{c}$ of ∼0.045.

**Figure 2:**
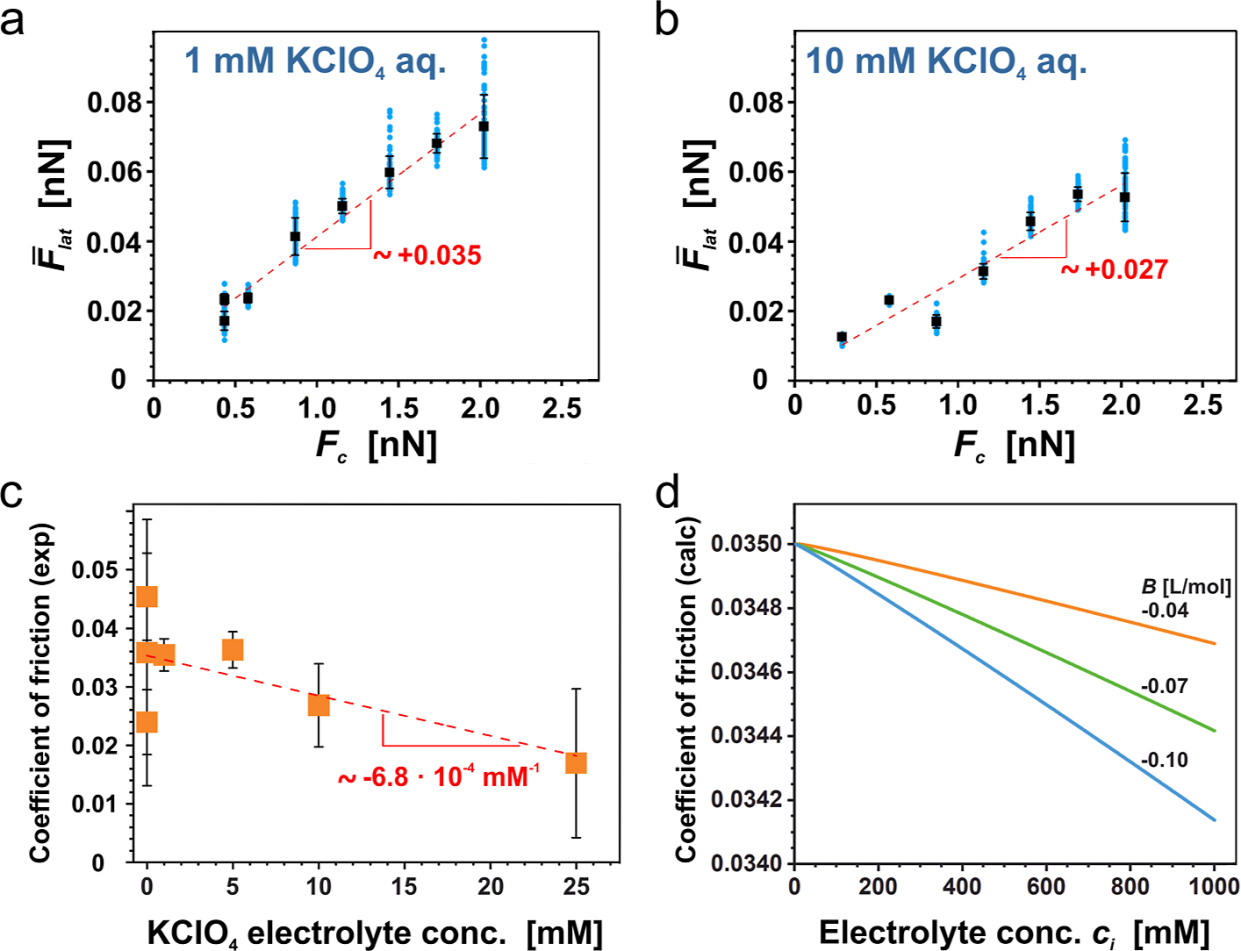
In situ AFM friction analysis for KClO_4_ electrolytes. (a,b) Lateral vs normal force plots for 1 mM and 10 mM KClO_4_, respectively. (c) Summary of resulting coefficient of friction (COF) values vs bulk electrolyte concentration, with linear regression (*dashed red* line). (d) COF variation with the electrolyte concentration *c*_*i*_ at the interface, as calculated from eq S4 μ_BL_ (*h* = 5.82, *l* = 0.25) for three negative values of the Jones–Dole *B*-coefficient.

As can be seen from the friction image (Figure 1b) of the polycrystalline gold surface in deionized water, the force level is relatively uniform. Expectedly, slightly higher friction is observed on intergranular regions (see also Figure S2b,d,f). Grain boundaries tend to show an increased surface defect density and also an enhanced electrocatalytic activity.^59^ In addition, the elevated friction may reflect the groove-like morphology, leading to an enlarged tip–sample contact area (potentially resulting in a higher measured friction force).

The coefficient of friction (COF) was obtained from the slope of lateral vs normal force plots, i.e. μ = $\Delta {\mathrm{F̅}}_{lat}/\Delta F_{c}$ , in deionized water (Figure 1d) and for aqueous KClO_4_ electrolytes at 1–25 mM (Figures 2a,b and S5). The summary plot (Figure 2c) shows that the experimentally determined friction coefficient, μ, decreases with increasing electrolyte concentration, indicating a lubricating effect. Accordingly, linear regression yields a negative rate of Δμ/Δ*c* ∼ −6.8 × 10^–4^ ± 4.8 × 10^–4^ (mM)^−1^. A similar trend is obtained using the arithmetic mean values of the friction loop’s trace and retrace friction plateaux (Figure S6) instead of the corresponding histogram peak positions determined by Gaussian fits (Figure S5).

Fundamental insight into complex ion effects can be gained from consideration of both interphasial ordering and ion-induced changes in the bulk electrolyte structure. Starting our analysis from the bulk electrolyte, where the water structure is dominated by ions rather than the interface, we adopt the semiempirical Jones–Dole equation (eq S3, Section S7), which describes the dependence of the relative dynamic viscosity η/η_0_ of an electrolyte on the molar concentration *c*.^60–62^ The Jones–Dole *B*-coefficient is a commonly used measure for the ion’s perturbation of the hydrogen-bond network:^20^ positive for kosmotropes (structure-making, e.g., Li^+^, Na^+^) and negative for chaotropes (structure-breaking, e.g., K^+^, Cs^+^).^61,62^ In the latter case, the thus calculated COF decreases with increasing electrolyte concentration *c*, as described in eq S4 (Supporting Information, Section S7) and shown in Figure 2d. The negative correlation in Figure 2c is qualitatively consistent with the chaotropic nature of K^+^ and ClO_4_^–^, which have *B* values of −0.009 and −0.058 L mol^–1^ at 25 °C, respectively.^61^ Rather than being a rigorous description of the junction between AFM tip apex and sample surface, the bulk viscosity consideration via the Jones–Dole relationship serves as a heuristic bridge to the nanoconfined interphase.

To rule out adhesion as the origin of the observed friction force variations (Figure 2c; eq S1, Section S7), force–distance curves were recorded for all electrolytes and the pull-off force, *F*_po_, was evaluated. Although specific ion adsorption could affect adhesion, it is known to be negligible for ClO_4_^–^ ions.^63–66^ As shown in Figure S8.I, the mean pull-off force is practically invariant (vanishing slope of ∼ 1.6 × 10^–3^ nN mM^–1^) over the 0–25 mM concentration range of aqueous KClO_4_ electrolytes (similarly to the trend obtained for the normalized adhesion force (Figure S8.III), using the experimentally determined tip radius (Figure S8.II)), thus indicating that the negative friction trend cannot be attributed to adhesion force changes.

Conceivably, a decrease in friction with increasing electrolyte concentration could arise from an ion-induced increase in the tip–sample distance, for example through the presence of K^+^ or ClO_4_^–^ ions in the nanoconfined gold/electrolyte/tip gap. Hydrated ions are larger than a water molecule (∼0.33 vs ∼0.28 nm for hydrated K^+^ and water, respectively),^67^ making this scenario plausible. Indeed, hydration lubrication by hydrated alkali metal cations has been reported as an effective friction-reduction mechanism in aqueous environments.^68^ In this regime, small ions with high charge density and large hydration numbers typically lead to stronger lubrication. In contrast, at high normal loads, friction can increase for ions with larger hydrated radii if higher activation energies are required to slide past each other.^67^ Experimental findings indicate distinct dissipation mechanisms in different force regimes: rate-activated hopping across energy barriers at high loads, shear deformation and relaxation of hydration shells at low loads.^67^ In the light of two different ion-dependent dissipation mechanisms, predicting friction behavior solely from the hydration lubrication concept remains challenging.

While the details of tip–sample distance variations and distance-dependent viscosity variations are complex and may involve specific ion effects, pronounced variations in interphasial viscosity can occur within hydration layers on hydrophilic surfaces and modulate the friction force acting on the scanning tip. Overall, our findings indicate that the observed decrease in friction ${\mathrm{F̅}}_{lat}$ (Figure 2c) cannot be explained solely by variations in the physical parameters commonly governing friction forces. Beyond elastic deformation and attractive force interactions, friction may also be influenced by the liquid medium surrounding the AFM tip. The arrangement of water molecules arises from hydrogen-bond interactions between water molecules but also from interactions of water with the solid surface,^69^ effects that are particularly pronounced under nanoconfinement. During the scanning motion, hydration layers between the tip and sample become disrupted and water molecules displaced. Consequently, the mechanical resistance exerted on the AFM tip decreases if the hydrogen-bond network is weakened or lubricating species are present. In fact, chaotropic alkali metal cations are known to soften the hydrogen-bond network, depending on the strength of their hydration shells. For K^+^ ions, similarly to ClO_4_^–^ ions, a mild chaotropic effect is expected (Section S7).

Importantly, effective ion concentrations at solid–electrolyte interfaces can deviate strongly from the nominal bulk values. In particular, interphasial ion concentrations at Au, Cu, and Pt electrodes can be enhanced over the bulk concentration by roughly a factor of ∼ 60–80.^3^ Effective values reflecting the interphasial ion concentration, *c*_i_, can be obtained by rescaling the nominal concentrations from Figure 2c. Assuming an enhancement factor of *f* = *c*_i_/*c* ∼ 70, the experimentally determined rate Δμ/Δ*c* (Figure 2c) changes by a factor of 1/*f* from ∼ −6.8 × 10^–4^ (mM)^−1^ to an effective rate of ∼ −9.7 × 10^–6^ (mM)^−1^, as the rate at the interface is given by Δμ/Δ*c*_*i*_ = Δμ/(*f* × Δ*c*)=(1/*f*) × Δμ/Δ*c*. This value can be compared to the rates derived from the COF vs concentration curves (Figure 2d) calculated from eq S4 at the concentration *c*_i_, i.e. μ_BL_ ≈ *h*(*U*η_0_(1 + *Ac*_i_^1/2^ + *Bc*_i_))^*l*^ with the Jones–Dole parameters *A*, *B* (eq S3 in Section S7), the sliding speed *U*, and the boundary friction coefficients *h*, *l* (eq S2).

As shown in Figure S9, the steepest curve yields −0.99 × 10^–6^ (mM)^−1^ at *B* = −0.1 L mol^–1^ and high concentrations (>500 mM). Since the experimentally determined slope (∼−9.7 × 10^–6^ (mM)^−1^) is about 1 order of magnitude larger, the friction reduction at the gold–electrolyte interface appears significantly stronger than predicted from the combined *B*-coefficient values for K^+^ and ClO_4_^–^ (adding up to ∼ −0.07 L mol^–1^).^61^ As this tentative comparison between predicted (eq S4) and measured COF variations involves assumptions (in particular, on the interfacial ion concentration *c*_i_), for quantitative analysis a more comprehensive model would be required that accounts for potentially occurring interfacial effects, in addition to the lubricating effect of chaotropic ions. In the context of the interfacial water structure, the chaotropic effect represents only one of several interactions potentially modulating the hydrogen-bond network of water. Other factors include the ordering of charged or polarizable electrolyte species, which rearrange in the presence of electrode surface charges or an applied electric potential, toward a state of lowest energy. AFM friction studies on hydrophobic surfaces suggested the presence of depleted hydration layers,^58^ whereas interfacial ordering effects nearby hydrophilic electrode surfaces typically exhibit a sharp rise in the interphasial viscosity within ∼ 1 nm of the surface.^4,18,19,23^ MD simulations of monovalent electrolytes further indicate that the viscosity in the outer Helmholtz layer increases strongly with the surface charge density.^70^ For the polycrystalline gold surfaces used here, the initial water contact angle of sessile droplets was ∼ 38° (Figure S10), confirming their hydrophilic character.

To explore the relationship between friction force variation and interphasial viscosity, we applied Feibelman’s model^71^ describing the lateral force acting on a parabolic-cylindrical tip moving across a planar surface, at a separation distance *D*. Importantly, the model assumes that each surface is covered by a hydration layer of thickness *w*, where the local viscosity exceeds that of the bulk solution (Figure S11a). The calculated lateral force (Figure S11b) increases sharply when the hydration layers of the two surfaces overlap (*D* < 2*w*), e.g., for *w* = 0.54 nm (corresponding to two hydration layers each). For a friction force of 0.036 nN (reflecting the case of *F*_c_ = 1 nN and a mean COF μ = 0.036 for neat water; Figure 2c), at a distance of *D* = 0.81 nm (∼ three water layers) the lateral force calculated from the model is equal for an interphasial viscosity of η_*i*_ of 14 × 10^4^ mPa s (Figure S11b). This value is roughly 5 orders of magnitude larger than the bulk viscosity of neat water (η_b_ ∼ 0.89 mPa s at 25 °C).^72^ Essentially, Feibelman’s model illustrates the effect of interphasial viscosity increase on the lateral force, while its assumptions are approximate. Hence, consideration of Feibelman’s model provides a link between the friction force analysis and the subsequent analysis based on MD simulations, which give a more detailed picture of interphasial viscosity variations.

To gain mechanistic insight, at a molecular level, into the effect of alkali metal cations on the interphasial viscosity variations, MD simulations of 0.5 and 5 molal NaCl, KCl and CsCl solutions were carried out, utilizing thermodynamically optimized force fields available for alkali metal and halide ions.^73,74^ For the simulated electrolytes, concentrations are reported as molalities, i.e. moles of solute per kg of solvent (500 mmol kg^–1^ solutions of NaCl, KCl and CsCl at molarities of 499.0, 496.8, and 497.4 mM, respectively; 5 mol kg^–1^ solutions at molarities of 4.612, 4.450, and 4.327 M). In the simulations, electrolytes are more highly concentrated than in our experiments so that the measured effects on viscosity are clearly distinguishable from statistical noise caused by the necessarily finite simulation time. Similarly to a gravity-driven flow, the liquid is pulled with a constant force that applies the same acceleration to each atom, parallel to a self-assembled monolayer (SAM) comprising decanol molecules (Figure 3a). Its OH- tail-groups’ partial charges are adjusted in such a manner that the SAM’s water contact angle (WCA) of 33° is similar to the gold films’ WCA (Figure S10 and Section S12). These driven-flow simulations yield interphasial liquid density (Figure S12.I), liquid velocity and surface–liquid stress profiles (Figure 3b), from which corresponding viscosity profiles can be calculated (Figure S12.III).^75^ Here, it is important to note that the surface–liquid friction force profile is spatially extended, acting over a distance of several Ångstrom along the surface–normal axis *z*. This spatially extended friction force is taken fully into account when calculating the viscosity profiles η­(*z*), which are shown in Figure 3c, compared with extracted *effective* viscosity profiles. This effective viscosity, η_eff_, would be the expected result of an experimental viscosity measurement where a no-slip boundary condition acting at a single position along the surface normal was assumed.^18,76,77^

**Figure 3:**
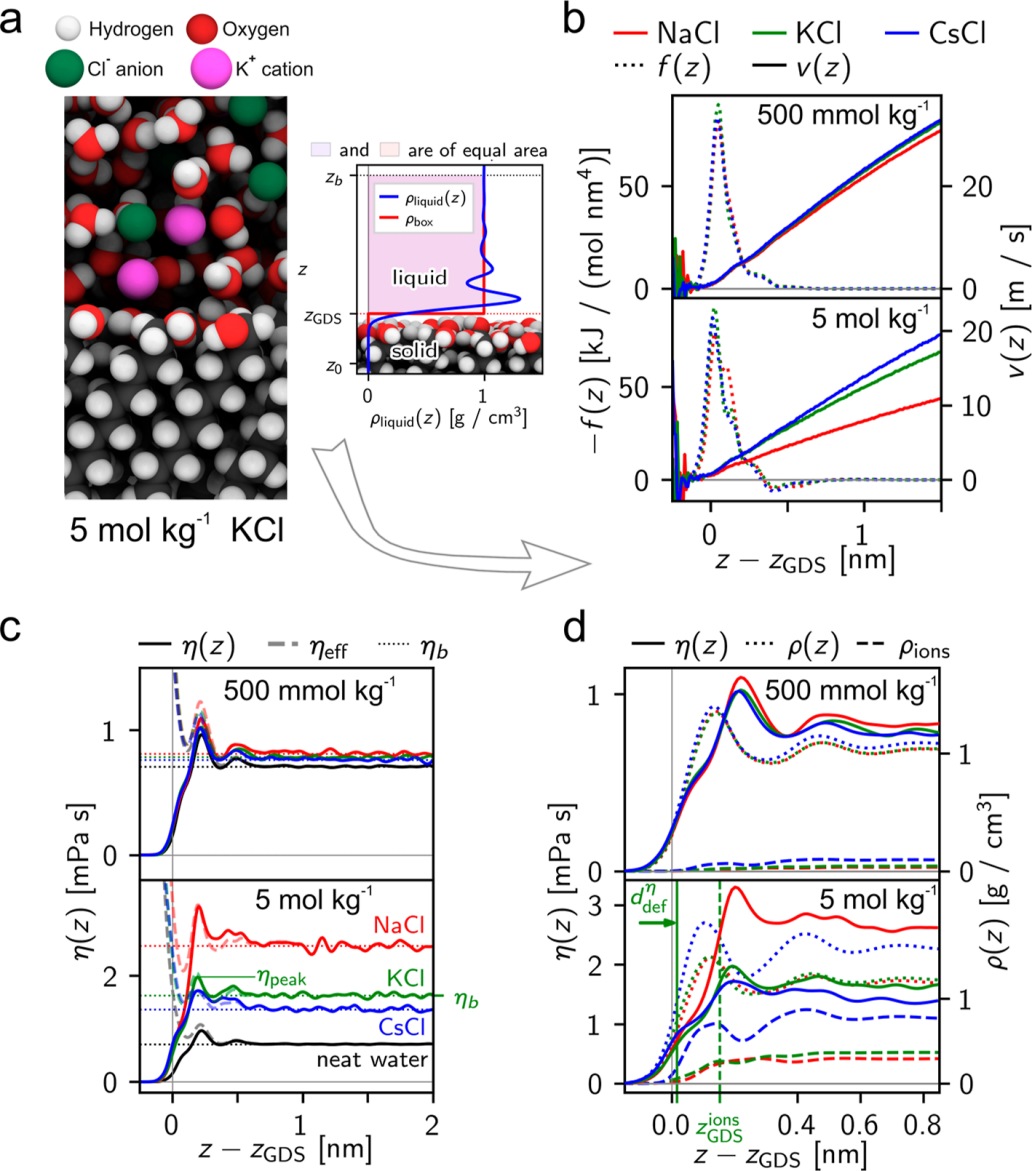
MD simulation analysis of the ion-specific position-dependent interphasial viscosity. (a) Snapshot of the interface between a 5 mol kg^–1^ KCl solution and a hydrophilic OH-terminated SAM. The corresponding schematic to the right shows how the Gibbs dividing surface (GDS) position, *z*_GDS_, is determined. (b) Velocity, *v*(*z*), and stress, −*f*(*z*), profiles vs distance from the GDS for gravity-like driven flow for 3 different electrolytes at concentrations 500 mmol kg^–1^ (*top*) and 5 mol kg^–1^ (*bottom*). (c) Interphasial viscosity profiles for NaCl, KCl and CsCl solutions at 500 mmol kg^–1^ and 5 mol kg^–1^ concentrations (*solid lines*), revealing characteristic interphasial viscosity profiles and different bulk viscosity levels. Corresponding extracted effective viscosity profiles are shown as *dashed* lines. (d) Close-up of the interphasial viscosity η­(*z*), electrolyte density ρ­(*z*) and ion density ρ_ion_, with the viscosity-deficit distance *d*_def_^η^ and the ion-deficit surface position *z*_def_^ions^ marked for the case of KCl.

Far from the interface, the viscosity profiles approach a bulk viscosity level, η_b_, that varies with the alkali metal cation type (Figures 3c and 4a). Zooming in on the first and second hydration layers near the interface in Figure 3d, it is apparent that the peaks for the ion density and especially viscosity profiles are shifted away from the interface. Essentially, the kosmotropic behavior expected for the Na^+^ ions leads to higher η_peak_ and η_b_ values, whereas the chaotropic behavior expected for the Cs^+^ ions is reflected by lower η_peak_ and η_b_ values. For K^+^, meanwhile the respective values lie between those for Na^+^ and Cs^+^, indicating moderate chaotropic behavior qualitatively similar to Cs^+^. In Figure 4a, the extracted bulk viscosities, η_b_, are collected and plotted over the average ion sizes (see ref 78 for compiled ion radii (nonhydrated); refs 79 and 80 for experimental values relating to NaCl and KCl/CsCl, respectively), with the trends among the different ion types agreeing well with values from literature. In Figure 4b, major characteristics of the viscosity profiles are analyzed as a function of the ion radius, *r*_ion_. While the plot of the viscosity peak heights, η_peak_, shows a behavior similar to the bulk viscosities (Figure 4a,b), i.e. a decrease with increasing *r*_ion_, the peak-position plot (Figure 4b *top*) displays the positional offset between viscosity and density peaks, showing significant variations for the case of 5 mol kg^–1^ electrolytes most of all.

**Figure 4:**
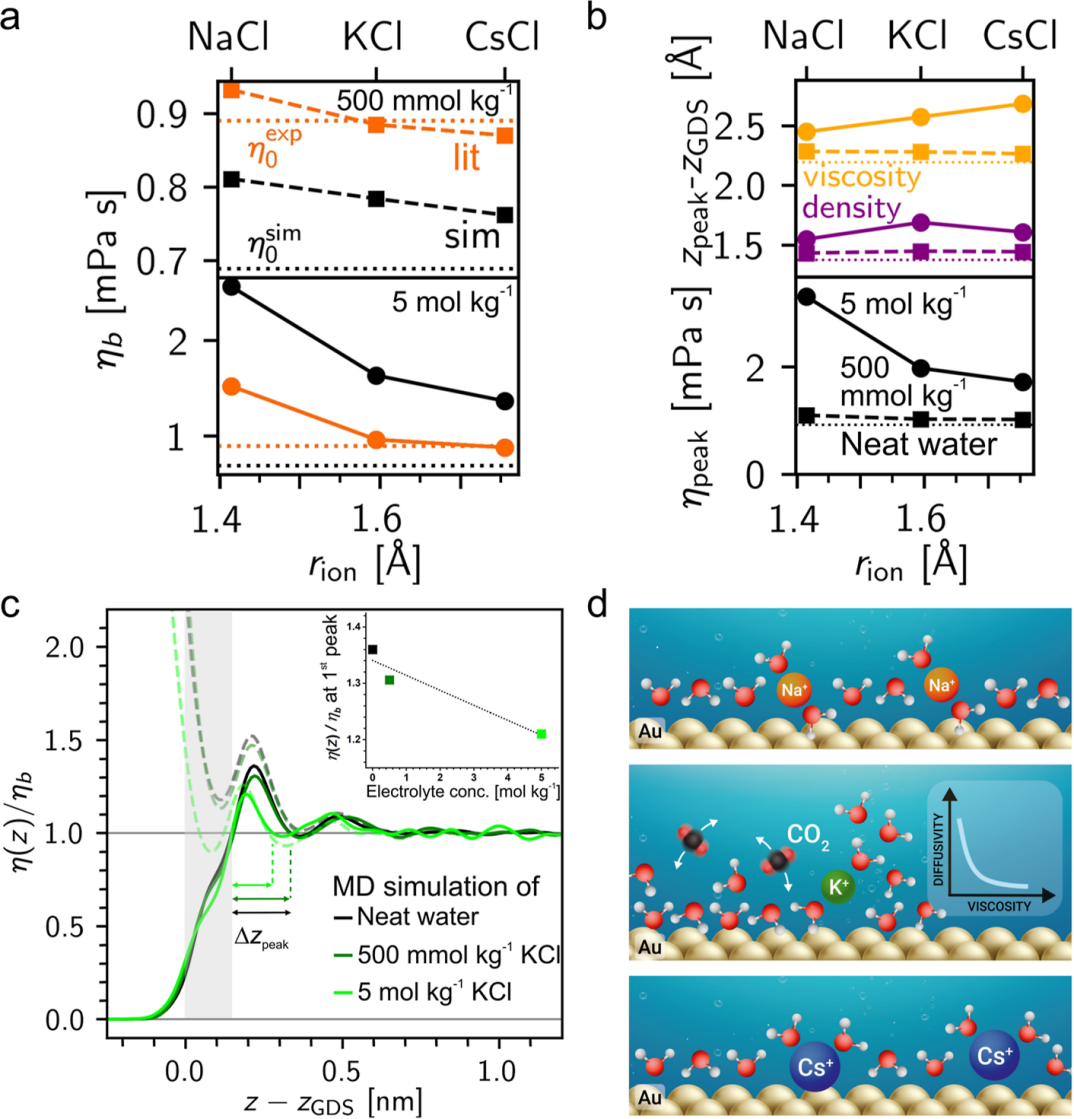
In-depth analysis of interphasial viscosity variations from MD simulations. (a) Bulk viscosity η_b_, vs the ion size from MD (black) compared to experimental values from literature (orange). (b) Position of viscosity and density peaks in η­(*z*), ρ­(*z*) and the viscosity value η_peak_ at its peak, for different ions and concentrations (500 mmol kg^–1^ and 5 mol kg^–1^—*dashed* and *solid* lines, respectively). (c) Profiles of the relative viscosity, η­(*z*)/η_b_ (solid lines), and its variation with the KCl electrolyte concentration (*dashed* lines—corresponding *effective* viscosity profiles). Inset: Plot of the first peak’s height (relative viscosity), showing its suppression with increasing concentration. (d) Interphasial hydration layer scenarios for Na^+^ (*top*), K^+^ (*middle*) and Cs^+^ (*bottom*), with the schematic for K^+^ also depicting the diffusivity (arrows *in white*) of dissolved CO_2_ molecules and its anticipated increase where the HB network structure is weakened by the chaotropic ion K^+^. Inset: Schematic plot of the diffusivity vs the viscosity η, indicating the ∝1/η relationship (Stokes–Einstein).

The apparent bulk-ward shift of the ion density and viscosity profiles (Figure 3d) indicates a surface deficit of ions that, in turn, entails a locally diminished viscosity. Notably, inside the deficit zone, the viscosity profiles each show a shoulder, which coincides approximately with the viscosity-deficit distance *d*_def_^η^, i.e. the displacement *z*_GDS_ – *z*_η_ of the viscosity dividing surfaces (where the liquid viscosity excess vanishes) away from the Gibbs dividing surface (GDS) of the respective liquid (where the mass density excess of the liquid vanishes).^75,81^ The *effective* viscosity profiles reflect the spatial variations of the interphasial viscosity and clearly show similar variations in the first peak between the deficit zone and the bulk electrolyte (Figure 4c). As the solid–electrolyte friction contributes to the effective viscosity, for *z* – *z*_GDS_ → 0 it increases rapidly for all three electrolyte types (Figure 3c).

The *d*_def_^η^ values are plotted in Figure S12.VI, over ion size (as reported in ref 61), together with the ion-deficit distances *d*_def_^ions^ (Figure S12.VI), which are the displacements along *z* of the ion density profile’s GDS from that of neat water. We observe a reduction in the surface viscosity-deficit distance from the neat water value of *d*_def_^η^ = 0.5 Å for all electrolytes. The 5 mol kg^–1^ CsCl electrolyte in particular, whose ions tend to reach closer to the surface than NaCl or KCl, shows a viscosity excess, with *d*_def_^η^ = −0.54 Å. To put these findings into context with the Jones–Dole *B*-coefficient, we recall that the latter becomes more negative with increasing alkali metal cation radius (Figure S13), i.e. the behavior of larger cations is more chaotropic. Hence, from the plot of *d*_def_^η^ (Figure S12.VI) it can be inferred that a more negative surface viscosity-deficit length is accompanied by a more negative *B*-coefficient.

As evidenced by the rise in the effective viscosity η_eff_ (Figure 3c), the reduction in local viscosity near the interface is compensated by the direct friction force with the interface. Clearly, in the region between the viscosity peak and the interface at *z* = 0 (Figure 4c, *gray-shaded* region) the effective viscosity deviates from the local viscosity. While the former shows a pronounced rise for *z* → 0 (interphasial friction zone), the latter decays to a vanishing value. Yet, the effective viscosity profile also shows a mild oscillatory behavior in the interphase region between the deficit and the bulk viscosity zones. Interestingly, normalized viscosity plots (Figure S12.V) indicate that the peak viscosities agree rather well among the different ions, i.e., the effect of the ions on the interphasial viscosity is nearly identical to their effect on the bulk viscosity.

Overall, this analysis clearly shows that it is paramount to consider local ion concentration, local surface friction and local viscosity, when aiming for a quantitative description of molecular mobility in the vicinity of a solid–liquid interface. Importantly, the interphasial viscosity profiles (Figure 3c,d), featuring a deficit zone as well as separate peaks rather than a mere exponential rise, provide a much more detailed insight, suggesting a hydration layer structure that varies with the presence of ions. Moreover, analysis of the viscosity peak heights and also the bulk viscosity values (Figures 3c,d and 4a,b) indicates that these characteristics are largely consistent with the chao-/kosmotropic behavior suggested by the Jones–Dole *B*-coefficient.

Given the pronounced interphasial viscosity peaks occurring in neat water, when in contact with a hydrophilic surface (Figure S12.II), it transpires that ion-induced modulation of the peak height can cause significant changes in the interphasial friction force. In fact, an empirical expression for the hydrodynamic friction coefficient has been suggested^56^ that features a friction term related to ion-induced reorganizing of the HB network and shows the highest value for the case of neat water. In terms of Feibelman’s model (Section S11), the viscosity peak can be tentatively approximated with the interphasial hydration layer of thickness *w*, where the local viscosity is increased compared to the bulk viscosity. Assuming the same hydration layer thickness for both sample and tip surfaces, the friction force is expected to rise significantly for mutual distances *D* < 2*w* (Figure S11b). Accordingly, the rise in friction should set in around a *D* value of ∼ 0.6 nm, for a distance of ∼ 0.3 nm from the GDS to the outer slope of the first interphasial viscosity peak (Figure 3d). It is interesting to note that the distance of ∼ 0.3 nm is comparable to one water molecular diameter of ∼ 0.28 nm.^67^

To rationalize the lubricating effect observed for the case of KClO_4_ aq. electrolyte (Figure 2c), we compare the viscosity profiles obtained from MD simulations for neat water and 500 mmol kg^–1^ and 5 mol kg^–1^ KCl. As can be seen from the plot in Figure 4c, with increasing electrolyte concentration, both the height (Inset of Figure 4c) and width of the first peak in the relative viscosity profiles decrease. A similar behavior is observed for the corresponding *effective* viscosity profiles where nonlocal surface–liquid friction effects are incorporated into viscosity effects (Figure 4c, *dashed* lines). While some changes in the position and height of the second viscosity peak can also be seen, these variations as well as those of the third and fourth peaks seem negligible.

Taken together, these findings indicate that the lubricating effect of chaotropic ions is closely linked to the interphasial viscosity profiles’ main characteristics, particularly the suppression of the first viscosity peak’s height and width. The decrease in peak height with increasing electrolyte concentration most likely reflects weakening of the hydrogen-bond network induced by chaotropic ions. This effect is stronger for Cs^+^ (Figure S12.V), as its hydration shell is relatively loosely bound. Unlike tightly hydrated Na^+^,^82^ Cs^+^ ions preferentially reside next to the gold–electrolyte interface (similar to specific adsorption within the Stern layer), accompanied by partial desolvation^83^ and local weakening of the interfacial hydrogen-bond network (Figure 4d). It should be noted that extrapolation of our MD simulation results to lower electrolyte concentrations is associated with some uncertainty due to possible ion–ion correlation and ion-crowding effects, but is in fact unavoidable because the ion-induced variations of the interphasial viscosity become indistinguishable from noise for smaller electrolyte concentrations.

Since the viscosity in a liquid at a planar surface becomes tensorial and depends on the separation from the interface, it is characterized by lateral and perpendicular viscosity profiles. An AFM tip probes predominantly the lateral viscosity if it is much larger than the liquid structural interfacial correlation length, which is less than 1 nm for water.^84,85^ Probing the vertical diffusivity of molecular motion is conceptually much more involved than probing the lateral diffusivity, as diffusive and free-energetic effects couple in the normal direction (out-of-plane). In previous theoretical work, it was demonstrated that lateral and normal water diffusivity profiles are rather similar, as shown for nonpolar and polar surfaces.^86^

As illustrated for K^+^, through the Stokes–Einstein relation linking viscosity and molecular mobility, interphasial viscosity variations can induce spatial variations in the diffusivity of reactants or intermediates involved in electrocatalytic reactions such as CO_2_RR (Figure 4d). Below the potential of zero charge, *E*_pzc_, cations further accumulate at the interface, with chaotropic ions (such as K^+^) weakening interfacial water organization and accumulating more readily than kosmotropic ions (such as Li^+^).^87^ At a potential of −0.9 V vs SHE, the interfacial K^+^ concentration was found to be about 74 times larger than the corresponding bulk concentration.^6^ Being highly concentrated in the Helmholtz layer, weakly hydrated cations (such as Cs^+^) that reside closer to the interface, modulate the interfacial electric field driving the adsorption of CO_2_. While the interfacial interactions of cations on electrocatalytic reactions encompass a variety of interrelated effects and can be concentration- and potential-dependent,^35^ at mild overpotentials alkali metal cations with a relatively low hydration energy (K^+^, Cs^+^)^24^ tend to be beneficial for the CO_2_RR reaction, among others due to their chaotropic effect.

Besides, it should be noted that specific transport mechanisms can occur, as is the case for proton transport by the Grotthuss mechanism,^88^ via tetrahedrally coordinated water. Since a highly ordered water structure is conducive for proton transport, for reactions relying on supply of H^+^ (such as HER under acidic conditions) a strengthened HB network is beneficial. In contrast, for reactions consuming H_2_O a robust HB network tends to slow down the migration of water molecules to the catalyst–electrolyte interface.^89^ Typically, the diffusive transport of molecules is described by local viscosity within a good approximation, as was shown for polar and nonpolar molecules.^90,91^ For proton-coupled electron transfer reactions relying both on the supply of protons and molecular reaction partners, the HB network should be neither very loose nor tight, to allow Grotthuss transport but also efficient diffusional motion of molecules moving through the viscous liquid. Therefore, for accurate microkinetic modeling of electrocatalytic reactions it seems crucially important to account for interphasial viscosity variations, rather than relying solely on the bulk viscosity value. The supply of reactants (e.g., CO_2_) to the catalyst–electrolyte interface may occur through diffusion parallel or perpendicular to the electrode surface, and for both directions electrolyte ions modulate the interphasial density as well as the viscosity profiles.

## 3 Conclusions and Outlook

Our results establish interphasial viscosity as a central descriptor linking ion-specific structuring of hydration layers to nanoscale transport. A manifestation of the viscosity profile variations with the electrolyte concentration is the experimentally observed lubrication effect, for the case of nanoconfined gold in potassium perchlorate (KClO_4_) aqueous electrolyte. The molecular dynamics simulation results show that chaotropic cations modulate the interfacial hydrogen-bond network, generating a near-surface low-viscosity zone followed by a decaying oscillatory profile with a prominent first peak whose magnitude and spatial extent scale with ionic radius. The resulting interphasial viscosity variations directly modulate molecular mobility, highlighting the critical role of nanoscale interfacial structuring in controlling transport and reaction kinetics.

In addition to bridging the science areas of lubricated friction and electrocatalysis, our synergistic approach further establishes a paradigm where ions are utilized for rational tuning of the interfacial microenvironment and molecular transport under nanoconfinement. Moreover, microkinetic models can provide a more accurate description by including interphasial viscosity variations, ideally alongside field-effects at the electrified interface. Beyond electrocatalysis, the impact of interfacial hydrogen bond network weakening by chaotropic ions extends to electrochemical sensing, where analyte transport to the electrode governs sensitivity and selectivity, particularly for large molecules. More broadly, these findings highlight how tuning of interphasial viscosity and hydration-layer organization can serve as a strategy to control molecular mobility and reactivity across electrochemistry, sensing, and nanofluidics.

## 4 Materials and Methods

### 4.1 Materials

A Si wafer (n-type doping, electric resistivity ∼ 15 ± 3 MΩ cm) with a 50 nm Au thin film on top of a 70 nm Ti film was obtained from MicroFabSolutions (Italy); the wafer chip size was ∼ 12 × 12 mm^2^. Prior to the AFM friction force measurements, the chips were rinsed with deionized water as well as n-propanol and, subsequently, exposed to a UV plasma (ZEPTO plasma cleaner by Diener electronic, Germany) for ∼ 150 s. KClO_4_ salt (*M*_W_ ∼ 138.55 g mol^–1^) of 99.99% purity or higher was purchased from Sigma-Aldrich. Water used for preparation of the electrolytes or cleaning purposes was deionized (resistivity 18.2 MΩ cm; Elga Purelab flex, Germany).

### 4.2 Atomic Force Microscopy

A Cypher ES AFM system (by Asylum Research/Oxford Instruments), fitted with an environmental scanner for imaging in liquid, was employed. The probe was mounted onto an EC AFM type cantilever holder using a clip, which was made of poly­(ether–ether–ketone) (PEEK) and served as a connector between the probe and the core component made of quartz glass. The sample was mounted onto a liquid cell fitted with a perfluoroelastomer O-ring. Prior to their assembly, the cell components and O-ring were rinsed using copious amounts of deionized water, acetone and n-propanol, with sonic agitation applied to the removable parts. Each rinsing step involved bidirectional blow-drying, using pressurized N_2_ gas.

AFM contact mode cantilevers with a high force sensitivity were used. The Si probes of type qp-Scont (by Nanosensors, Switzerland) had a rectangular beam geometry with a typical spring constant of ∼ 0.011 nN/nm. The nominal tip height and the radius of curvature were ∼7 μm and ∼10 nm, respectively. Prior to mounting a cantilever, it was cleaned by soaking in deionized water for ∼20 min and subsequent exposure to a UV plasma for ∼90 s. For reference, Si_3_N_4_ probes of type XNC-A (by MikroMasch, Bulgaria) were used that had a triangular beam geometry with a typical spring constant of ∼0.08 nN/nm, a tip height of ∼3.5 μm and a nominal tip apex radius of ∼10 nm.

The cantilever spring constant was determined by the thermomechanical method^92^ (as implemented in the AFM control software), combined with measurement of the optical deflection sensitivity via force–distance curves on a Si wafer. For lateral/friction force calibration, a TGF11 type calibration grating (by MikroMasch) with sloped surface segments was scanned that lends itself to the wedge method,^93^ which gives the overall calibration constant.^57^ To account for the different refractive indices of air (for the case of calibration measurements) and aqueous electrolytes, the calibration constant values were rescaled by a factor *n*_air_/*n*_aq_ ∼ 1/1.36.^94^

The lateral/friction force measurements were carried out in lateral force microscopy (LFM) mode, at open-circuit potential. Friction loops, at a scan range of 600 nm and a scan speed of ∼ 1.8 μm s^–1^, were measured for a range of normal forces applied by the AFM cantilever. For each normal force value, about 100 friction loops were recorded, at several positions. The lateral force was determined from the loop half-width, a common procedure for eliminating lateral force components originating from topographic slope variations.^57,93,95^ To this end, the signal levels over the two friction loop segments, measured in trace and retrace, were determined both from the arithmetic mean and by fitting a Gaussian function to the corresponding histograms of lateral force signal values. Python 3.7.6 under the IDE Spyder 4.1.2 was employed for processing of friction data. The software packages AtomicJ^96^ (version 1.7.2) and Gwyddion^97^ (version 2.55, by the Czech Metrology Institute) were employed for processing and analysis of AFM force–distance curves and images, respectively. OriginPro 2019b, version 9.6.5.169, was employed for general data analysis purposes including plotting and linear regression.

### 4.3 Electrochemistry

For determination (Section S4 in the Supporting Information) of the potential of zero charge, *E*_pzc_, the gold film was connected to a SP-300 type potentiostat (by Bio-Logic Science Instruments). A silver wire and a platinum ring were mounted onto the AFM liquid cell that served as the reference and counter electrodes, respectively. Employing the associated EC-Lab control software, cyclic voltammetry curves were run over the range −500 to 500 mV, at sweep rates of 2.5 or 5.0 mV s^–1^, and chronoamperometry curves were recorded at various potentials nearby the expected *E*_pzc_ value.

### 4.4 Water Contact Angle

Individual sessile droplets of ∼ 25 to 50 μL (initial volume) deionized water were dispensed on the freshly cleaned gold film, mounted onto the sample platform of an OCA50 contact angle station (by dataphysics) and imaged from the side, employing the computer-controlled digital camera. The room temperature and relative humidity were ∼ 26 °C and 28%, respectively.

### 4.5 X-ray Diffraction

For ex-situ crystallographic analysis of polycrystalline gold films, a Bruker D8 Advance diffractometer in thin film (parallel beam) configuration was employed.

### 4.6 Scanning Electron Microscopy

For SEM imaging of the AFM tips, a Hitachi S-4800 system including a cold field emission gun was employed, allowing for acceleration voltages in the range from 0.1 to 30 kV. It was fitted with separate detectors for backscattered, secondary and transmitted electrons.

### 4.7 Molecular Dynamics Simulation

Force-field molecular dynamics (MD) simulations of 200 ns duration were carried out in GPU-enabled single-precision GROMACS 2023.3.^98,99^ The velocity rescaling (CSVR) thermostat was applied to all atoms with a target temperature of 300 K.^100^ Lennard-Jones forces were modeled with force-switching between 1.9 and 2.0 nm. Electrostatic forces were modeled using particle-mesh Ewald beyond a real-space cutoff of 2 nm. The simulated system was a decanol-SAM surface with an adsorbed electrolyte slab in an 8.946 × 6.887 × 40 nm box with periodic boundaries. The SAMs consisted of 288 decanol molecules. The bottom carbon of each molecule was restrained to a fixed point in space. These points were arranged in a close-packed hexagonal lattice in the *x*–*y*-plane with a nearest-neighbor distance of 4.97 Å.^101–103^ The OH-group partial charges of the decanol molecules were reduced by 10%, giving a WCA for the surface of 33°, which was chosen to be near the WCA of gold of 38°. The liquid consisted of 8192 water molecules and the necessary number of anion–cation pairs to achieve the target salt concentration. Above the liquid was vacuum in which a vapor phase could form. SAM molecules were modeled using the OPLS All-Atom (OPLS-AA) force field.^104–106^ Water was modeled using the SPC/E water model,^107^ and solvated ions were modeled using force fields optimized for electrolytes for a wide range of concentrations.^73,74^ A gravity-like driving force of 800 kJ/(mol nm^2^), which corresponds to a stress of 21.563 MPa, was applied in the *x*-direction to the liquid center of mass, i.e. a force was applied to each atom that is proportional to its mass. In the bulk, where the liquid density is constant, this gave a constant force density, and (ideally) a quadratic flow profile with the vertex of the parabola at the liquid–vapor interface. For the simulated electrolytes, concentrations are reported as molalities, i.e. moles of solute per kg of solvent (see Section S12 in the Supporting Information where a more detailed description of the simulation methods can be found). While the absolute peak height and position of viscosity profiles are somewhat dependent on the applied smoothing (Figure S12.IV), we note that the same procedure was used for all systems (convolution of initial velocity profile with a Gaussian kernel given by σ_sm_ = 0.4 Å).

## Supplementary Material



## Notes

Crystallographic analysis of the polycrystalline gold films; lateral force microscopy imaging of a polycrystalline gold surface in water; basics of AFM friction force measurement; potential of zero charge of the polycrystalline gold surface; coefficient of friction for the electrolytes 5 and 25 mM KClO_4_ aq. (from Gaussian fits to histograms of friction loop plateaux); coefficient of friction variations with the electrolyte concentration (from the arithmetic friction loop analysis); Jones–Dole’s relative viscosity in the context of boundary lubrication; adhesion force analysis; rates of the coefficient of friction variations resulting from the Jones–Dole relationship; water contact angle of the polycrystalline gold surface; hydration layer effects in a tip–sample configuration as described by Feibelman’s model; interphasial viscosity profiles from molecular dynamics simulation; Jones–Dole *B*-coefficient versus ion radius; related references (PDF)
