# Supplementary material for: Cation-Induced Interphasial Viscosity Variations on Gold Electrocatalysts in Nanoconfined Aqueous Electrolytes
Source: J Am Chem Soc. 2026 Jun 12;148(25):26353–64. doi: 10.1021/jacs.6c06065 (PMC13339140; doi:10.1021/jacs.6c06065)
Supplement: Supplementary file 1 [file ja6c06065_si_001.pdf]

Supporting Information for

Cation-induced interphasial viscosity variations on gold electrocatalysts in nanoconfined aqueous electrolytes

Martin Munz,[1,2] Shane Carlson,[3] Leon Jacobse,[2] Roland R. Netz,[3*] Beatriz Roldan-Cuenya,[2] Christopher S. Kley[1,2*]

[1] Helmholtz Young Investigator Group Nanoscale Operando CO_2 Photo-Electrocatalysis, Helmholtz-Zentrum Berlin für Materialien und Energie GmbH, 14109 Berlin, Germany

[2] Department of Interface Science, Fritz Haber Institute of the Max Planck Society, 14195 Berlin, Germany

[3] Fachbereich Physik, Freie Universität Berlin, Arnimallee 14, 14195 Berlin, Germany

*Corresponding authors. Email: christopher.kley@helmholtz-berlin.de, rnetz@physik.fu-berlin.de

Contents

S1. Crystallographic analysis of the polycrystalline gold films

S2. Lateral force microscopy imaging of a polycrystalline gold surface in water

S3. Basics of AFM friction force measurement

S4. Potential of zero charge of the polycrystalline gold surface

S5. Coefficient of friction for the electrolytes 5 and 25 mM $KClO_4$ aq, from Gaussian fits to histograms of friction loop plateaux

S6. Coefficient of friction variations with the electrolyte concentration, from the arithmetic friction loop analysis

S7. Jones-Dole's relative viscosity in the context of boundary lubrication

S8. Adhesion force analysis

S9. Rates of the coefficient of friction variations resulting from the Jones-Dole relationship

S10. Water contact angle of the polycrystalline gold surface

S11. Hydration layer effects in a tip-sample configuration as described by Feibelman's model

S12. Interphasial viscosity profiles from molecular dynamics simulation

S13. Jones-Dole *B*–coefficient *versus* ion radius

References (Supporting Information)

S1. Crystallographic analysis of the polycrystalline gold films

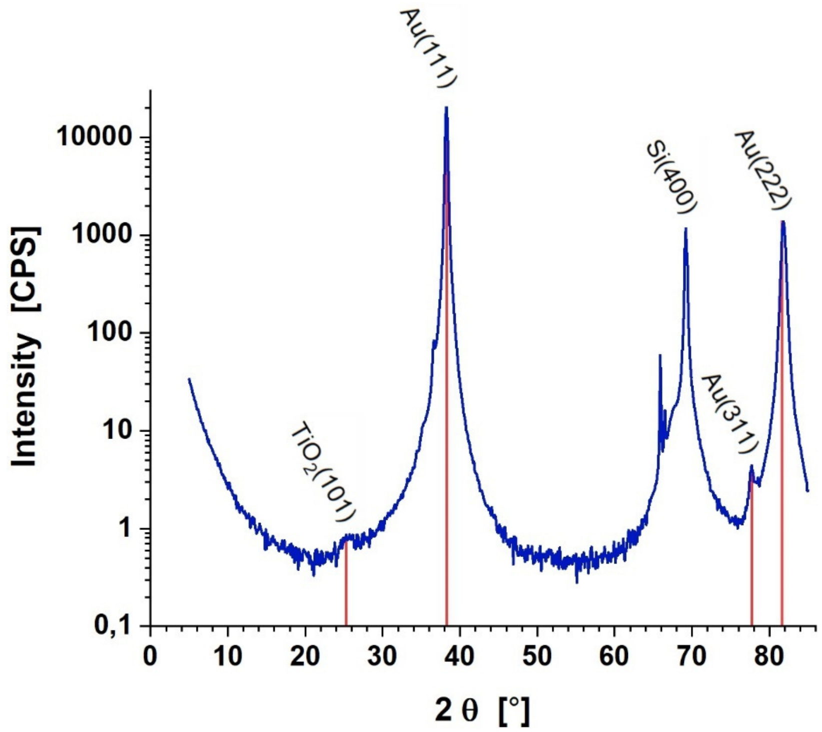

Figure S1. X-ray diffractogram of a polycrystalline gold film. Clearly, Au(111) is the predominant crystallite orientation. Further to peaks related to gold, i.e. Au(111), Au(222) and Au(311), a weak TiO_2(101) peak originating from the Ti sublayer can be seen. The Si(400) peak results from the Si wafer substrate.

S2. Lateral force microscopy imaging of a polycrystalline gold surface in water

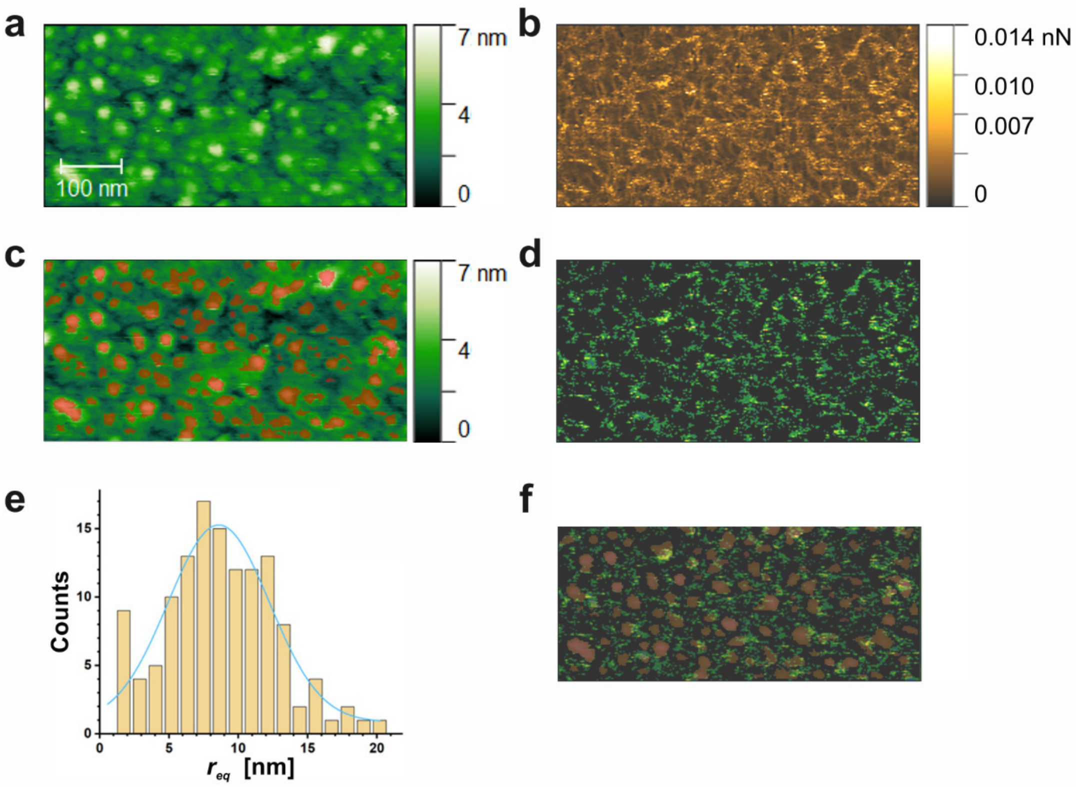

Figure S2. Lateral force microscopy (LFM) imaging of the polycrystalline gold surface, in deionized water. **a** Height image (scan area 600 x 300 nm^2), as obtained by contact mode AFM at a load of ~0.64 nN and a scan speed of 1.8 µm s^{-1}. The RMS roughness, S_q, is ~0.90 nm. **b** Friction force image. **c** Individual grains marked by the Watershed segmentation method. **d** As the friction force image in b, but with highlighted contrast. **e** Grain size distribution as resulting from c, in terms of the equivalent disc radius r_{eq}. **f** Overlay of the images shown in c and d.

S3. Basics of AFM friction force measurement

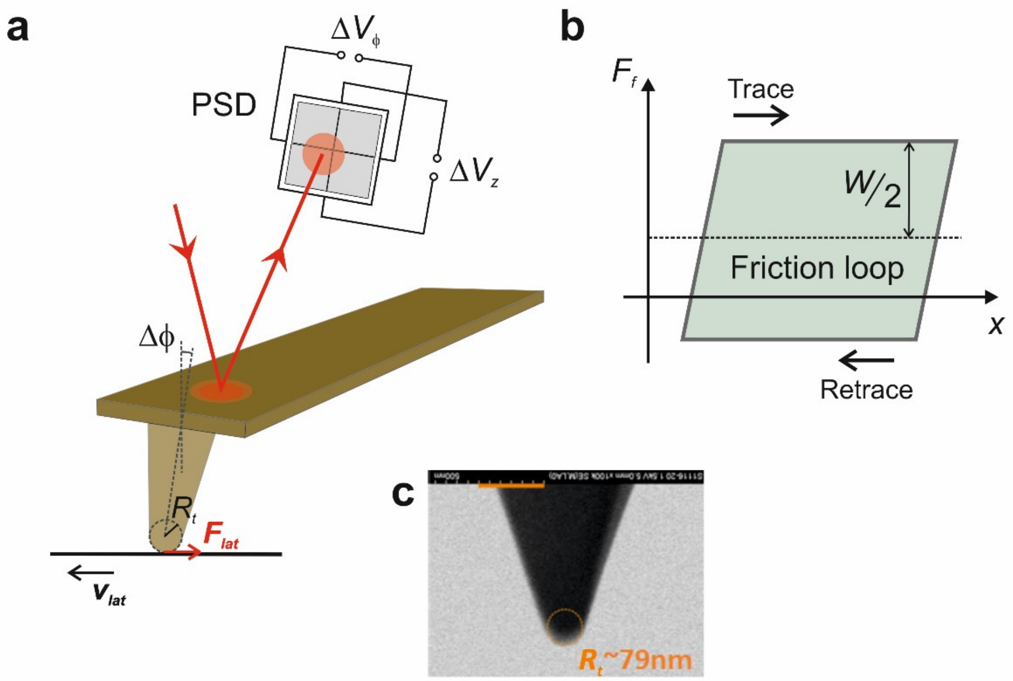

Figure S3. Schematic representation of the lateral force microscopy (LFM) approach. **a** Upon scanning motion at velocity v_{lat} , a friction force acting on the tip-sample contact causes a torsional cantilever deformation that is detected *via* the deflection of the reflected laser beam, resulting in a voltage readout ΔV_ϕ at the position sensitive diode (PSD). **b** A friction loop of width W is recorded by scanning back and forth over a certain distance while the tip is in permanent contact with the sample surface and the normal force applied by the cantilever is kept constant by the feedback loop for height imaging. **c** SEM micrograph of an AFM tip with a view from the side, for determination of the approximate tip radius R_t (here, R_t~79 nm).

With the AFM tip acting as a torsional arm, a friction force in the tip-sample contact causes a torsional cantilever deformation that is read out *via* a displacement of the reflected laser beam on a position sensitive diode (PSD, signal ΔV_ϕ), similarly to the detection of the cantilever deflection caused by normal forces (signal ΔV_z). The friction force is given by the friction loop half-width [1], whereas contributions originating from topographic slope variations are eliminated [1], [2]. Similarly, in lateral force microscopy (LFM) a friction force image is obtained from the difference of torsional signal images recorded in trace and retrace.

S4. Potential of zero charge of the polycrystalline gold surface

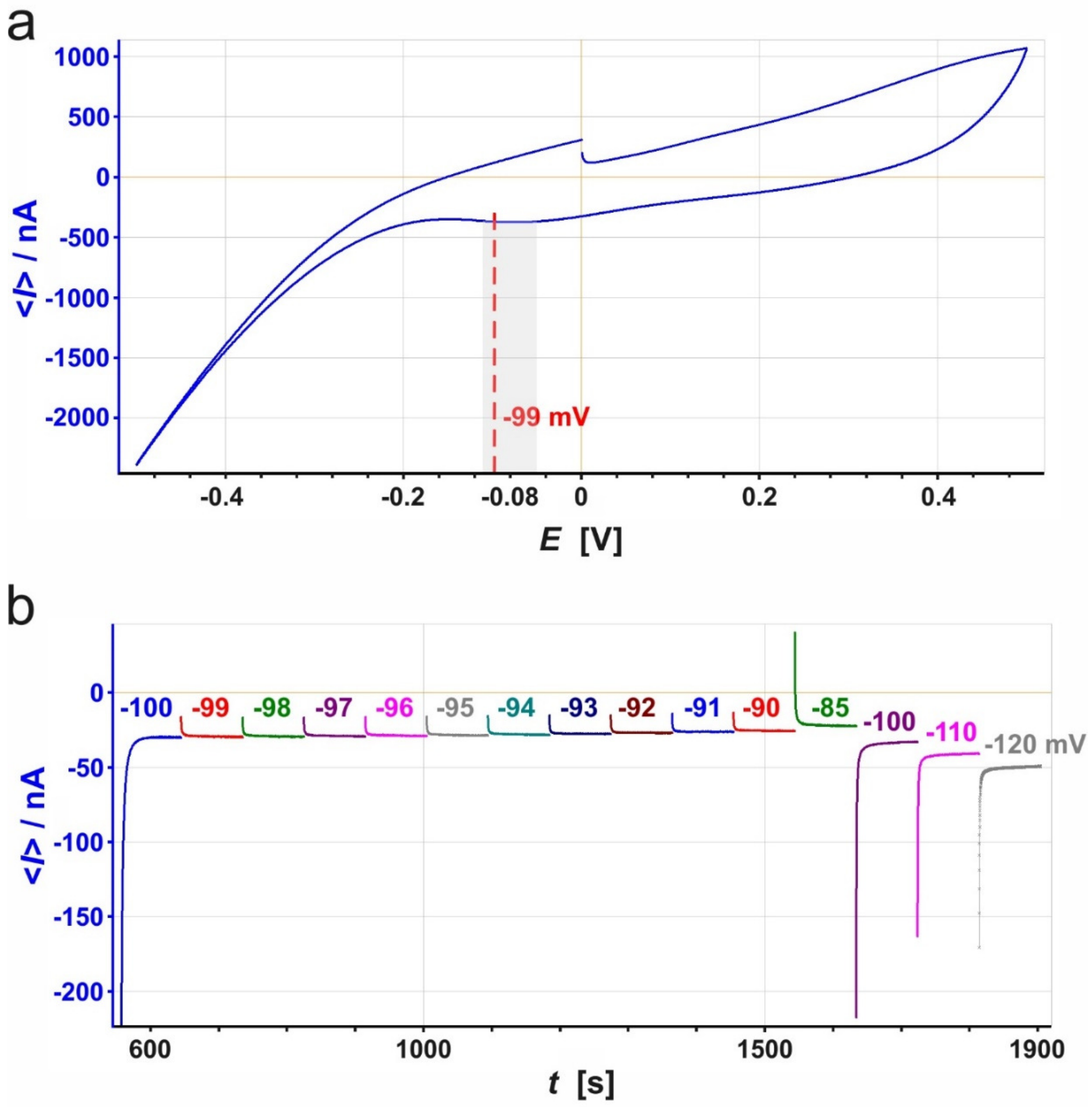

Figure S4. Potential of zero charge, E_{pzc}, of the polycrystalline gold surface in deionized water. **a** Cyclic voltammetry curve showing a dip-like feature around ~-80 ± 30 mV vs. Ag (cathodic scan; *grey* shaded area). **b** A series of chronoamperometry curves, measured at various potentials E_i. As the curve with the lowest initial current (amount) is the closest to E_{pzc} [3], the curve measured at -99 mV vs. Ag signifies E_{pzc}. Corresponding values vs. Ag/AgCl are approximately given by $E[\text{Ag/AgCl}] \approx E[\text{Ag}] + 0.05\ \text{V}$.

S5. Coefficient of friction for the electrolytes 5 and 25 mM $KClO_4$ aq, from Gaussian fits to histograms of friction loop plateaux

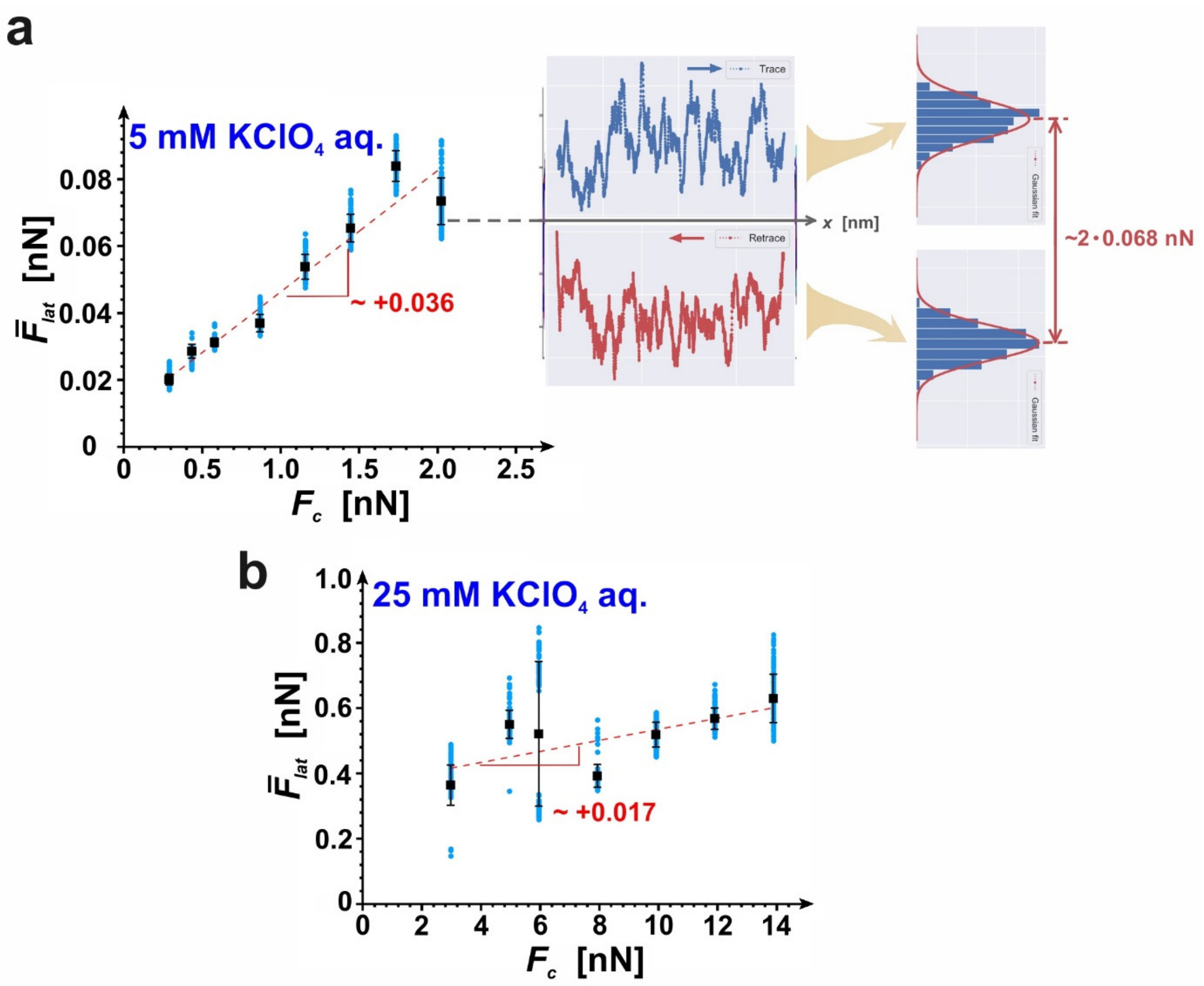

Figure S5. *In situ* lateral force measurements for various concentrations of the $KClO_4$ aq. electrolyte. **a** Plot of $\overline{F}_{lat}$ vs. F_c, for the case of 5 mM $KClO_4$ aq. electrolyte. *Inset:* The data analysis approach involving Gaussian fits to the histograms of both plateaux (trace/retrace) is indicated for an individual friction loop. **b** Plot of $\overline{F}_{lat}$ vs. F_c, for the case of 25 mM $KClO_4$ aq. electrolyte.

S6. Coefficient of friction variations with the electrolyte concentration, from the arithmetic friction loop analysis

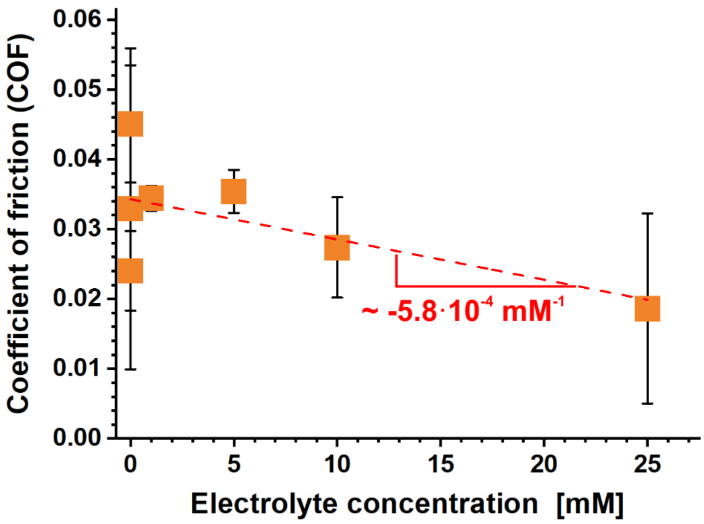

Figure S6. Plot of the coefficient of friction (COF) versus the concentration of aqueous $KClO_4$ electrolytes. The COF values (squares, in *orange*) resulted from the slopes $\Delta\overline{F}_{lat}/\Delta F_c$ of $\overline{F}_{lat}$ -vs- F_c plots (similar to Figs. 1d, 2a-b). The linear fit (dashed line, in *red*) shows a negative trend (rate $\Delta\mu/\Delta c$ ~-5.8·10^{-4} mM^{-1}).

S7. Jones-Dole's relative viscosity in the context of boundary lubrication

According to Amontons' law, the friction force, F_{lat}, acting on a sliding tip (Figs. 1a and S3a) scales in a linear manner with the total normal force, F_n, comprising the normal force applied by the AFM cantilever, F_c, and the attractive force, F_a, acting between the tip and the sample surfaces:

$$F_{lat} = \mu F_n = \mu(F_c + F_a) \,, \tag{S1}$$

with the coefficient of friction, μ.

When scanning in an aqueous solution, the coefficient μ of Amontons' linear relationship between friction force, F_{lat}, and F_n, is given by the coefficient μ_{BL} describing the friction in the boundary lubrication regime (Fig. S7), which can be written in terms of a power law as derived empirically by Bongaerts *et al*. [4]:

$$\mu_{BL} = h(U\eta)^l \,, \tag{S2}$$

where h and l are the power-law coefficient and exponent, respectively. The product of sliding speed, U, and the dynamic viscosity, η, of the liquid medium is reminiscent of Stribeck plots, where the coefficient of friction is analysed across the boundary, mixed and hydrodynamic lubrication regimes (Fig. S7). A Stribeck curve shows the friction coefficient versus the Hersey (Sommerfeld) number, a dimensionless lubrication parameter given by $U\eta/P$ (with the average load P) [5].

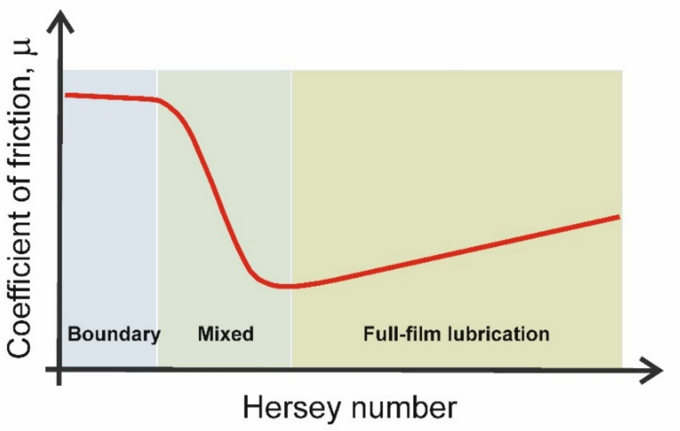

Figure S7. Schematic of a Stribeck curve describing the change in friction coefficient, μ, with the Hersey number. The curve valley signifies the transition between the mixed and full-film lubrication regimes. The boundary lubrication regime occurs for low values of the Hersey number, particularly for low viscosities. Adapted from Fig. 1 in Ref. [5], "Development of a Set of Stribeck Curves for Conformal Contacts of Rough Surfaces", *Tribology Transactions* 49 (2006) 526, Taylor & Francis.

Fundamental insight into complex ion effects can be gained both, from consideration of interfacial ordering and from ion-induced variations in the bulk electrolyte structure or related properties. In a scenario where the water structure is dominated by the presence of ions rather than the interface, we consider the relative dynamic viscosity η/η_0 of an electrolyte of the molar concentration c, as given by the semi-empirical Jones-Dole expansion [6–8]:

$$\frac{\eta}{\eta_0} \approx 1 + Ac^{1/2} + Bc \quad , \tag{S3}$$

where η_0 denotes the viscosity of the neat solvent. While the coefficient A (in $L^{1/2}\,mol^{-1/2}$) depends on the ion-ion interaction, the coefficient B (in $L\,mol^{-1}$) reflects ion-solvent interactions and is related to the ion volumes. The Jones-Dole $B-$ coefficient quantifies the degree to which the ion causes a perturbation to the adjacent network of HBs [9]. For kosmotropes (i.e. water structure-making ions, such as Li^+ and Na^+) the $B-$ coefficient for electrolyte bulk viscosity is positive, whereas for chaotropes (i.e. water structure-breaking ions, such as Cs^+) it is negative [7], [8]. Concerning the $B-$ coefficient, values of −0.009 and −0.058 $L\,mol^{-1}$ are given for K^+ and ClO_4^-, respectively [7], thus indicating chaotropic behaviour.

Hence, insertion of Eq. (S3) into Eq. (S2) for the coefficient of friction, μ_{BL}, in the boundary lubrication regime (Fig. S7) yields an expression including the $A-$ and $B-$ coefficients of the Jones-Dole expansion:

$$\mu_{BL} \approx h\left(U\eta_0\left(1 + Ac^{1/2} + Bc\right)\right)^{l} \quad . \tag{S4}$$

In the case of a negative $B-$ coefficient, μ_{BL} decreases with increasing concentration c of the electrolyte (Fig. 2d). Thus, Eq. (S4) casts the rationale for the lubricating effect of chaotropic ions into a formal description.

S8. Adhesion force analysis

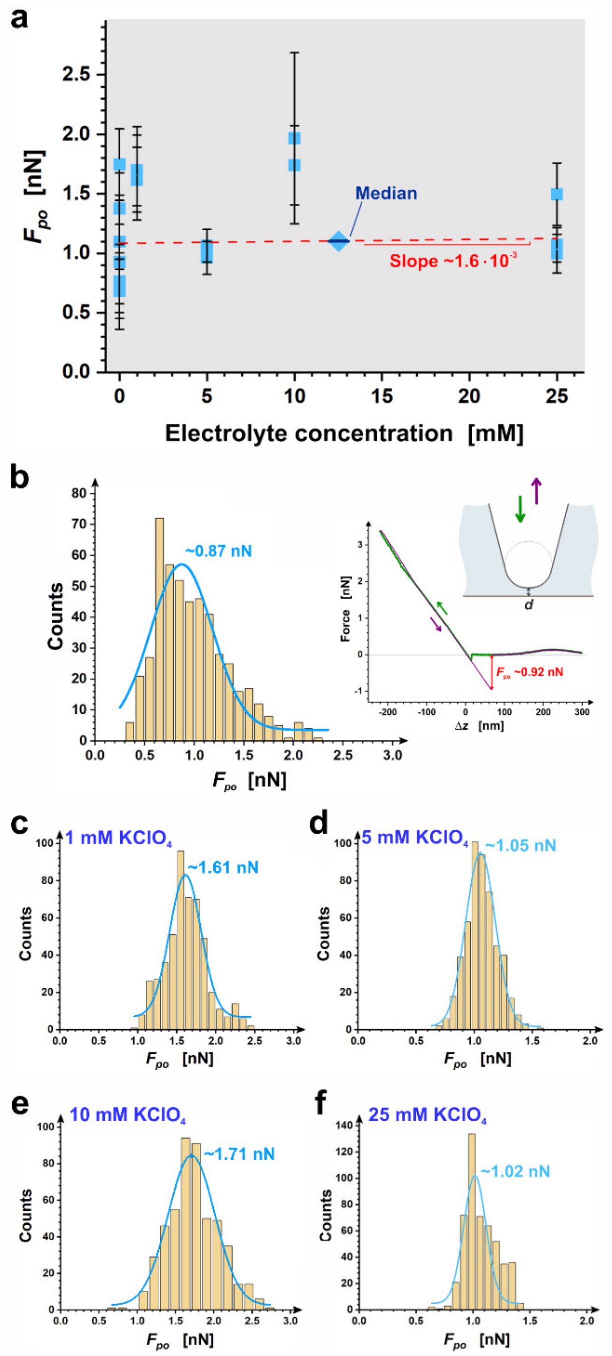

Figure S8.I. Demonstration that adhesion force variations with the electrolyte concentration are negligible. **a** Plot of the mean pull-off force, F_{po}, values *vs*. the concentration of $KClO_4$ electrolytes. **b** Histogram of pull-off force values, for the case of deionized water. *Inset*: A force-distance curve and a schematic of its measurement. **c-f** Histograms, for the cases of 1, 5, 10 and 25 mM $KClO_4$ aq. electrolytes. All force-distance curves of this series were measured using the same AFM cantilever, type qp-Scont, with an approximate tip radius of R_t ~79 nm.

On average, the pull-off force was ~1.27 ± 0.41 nN (median ~1.10 nN). Accounting for the tip radius of curvature of R_t ~79 nm determined from SEM micrographs (Figs. S3c and S8.II), the normalised pull-off force was F_{po}/R ~0.016 ± 0.005 nN·nm^{-1} ($\left(F_{po}/R\right)_{\mathrm{median}}$ ~0.014 nN·nm^{-1}; Fig. S8.III a). For the case of DMT contact mechanics [10], $F_{po}/R \sim 2\pi\cdot\gamma$ and $\left(F_{po}/R\right)_{\mathrm{median}}$ gives a value of ~2.2 mJ·m^{-2} for the interfacial energy γ, in excellent agreement with work of adhesion values, at the E_{pzc}, reported for gold-mica interfaces in the presence of 1 to 10 mM $KClO_4$ aqueous electrolytes [11].

Notably, a similarly low adhesion force variation over the range of $KClO_4$ concentrations was observed for the case of the Si_3N_4 tip (Fig. S8.III b). Hence, the vanishing trend of the plot in Fig. S8.I a (and correspondingly in Fig. S8.III a) does not seem specific to Si tips, which can carry a negative surface charge for a wide range of electrolyte pH > 3 due to the deprotonation of surface silanol groups (SiOH) or trapping of OH^- ions of silica [12–14]. While in the case of Si_3N_4 hydrolysis can occur with elution of ammonia and initial formation of silica at the solid surface [15], the surface chemistry is somewhat different but still involves silica species.

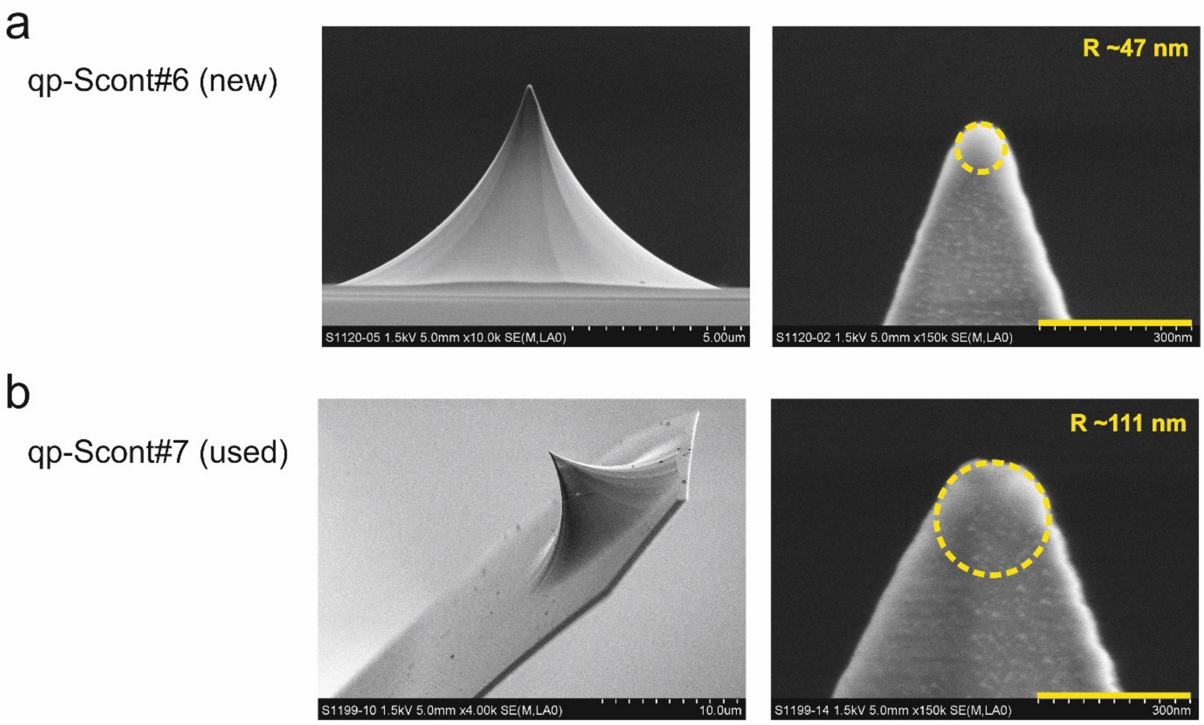

Figure S8.II. SEM micrographs of qp-Scont type AFM cantilevers used for *in situ* LFM. The approximate tip apex radii were determined from inscribed circles, as indicated by the dashed orange lines. **a** Overall view and a close-up of a new Si tip, i.e. imaged prior to AFM friction force measurements. **b** Another Si tip, as imaged after a series of AFM friction force and force-distance curve measurements.

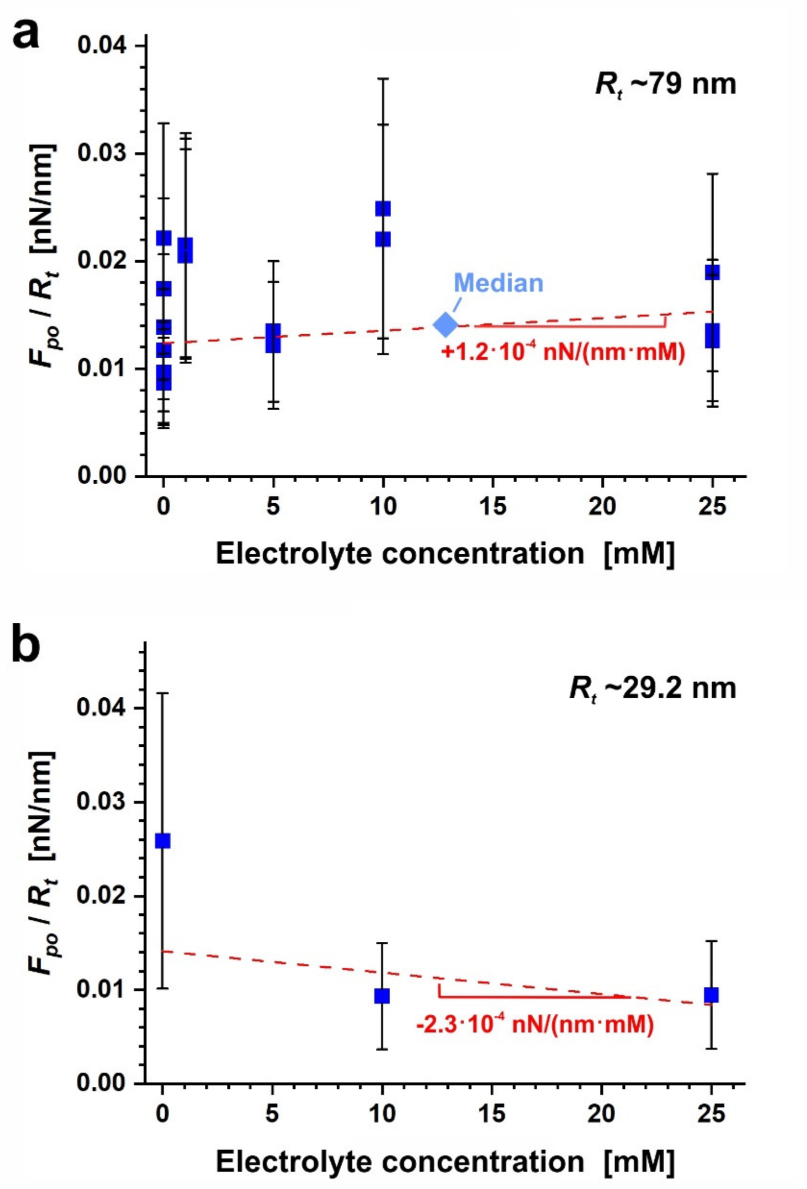

Figure S8.III. Plots of the normalized adhesion force vs. the concentration of $KClO_4$ aq. electrolytes. **a** As Fig. S8.I a, however accounting for the tip radius, R_t. The adhesion is given by the pull-off force F_{po} extracted from force-distance curves, and the force values are normalized by R_t. **b** Adhesion force variation, for the case of a Si_3N_4 tip. All force-distance curves of this series were measured using the same AFM cantilever, type XNC-A, with an approximate tip radius of R_t ~29.2 nm.

Further to the Au-electrolyte interface, also the electrolyte-SiO_x interface deserves attention. The SiO_x surface chemistry of the scanning Si tip involves its own hydration layer, facing the gold film's hydration structure. To investigate whether the adhesion force behaviour changes with the tip surface chemistry, a similar series was measured using a Si_3N_4 tip (Fig. S8.III b).

As a similarly low adhesion force variation over the range of $KClO_4$ concentrations was observed for the case of the Si_3N_4 tip (Fig. S8.III b), the vanishing trend of the plot in Fig. S8.I a (and correspondingly in Fig. S8.III a) does not seem specific to Si tips. Notably, compensation of negative surface charges by accumulation of (partially hydrated) cations occurs gradually with increasing ion concentration; and neutralization needs concentrations in the range of ~100 mM to ~1 M (depending on the AMC type and pH [16]), i.e. at much higher concentrations than 25 mM.

S9. Rates of the coefficient of friction variations resulting from the Jones-Dole relationship

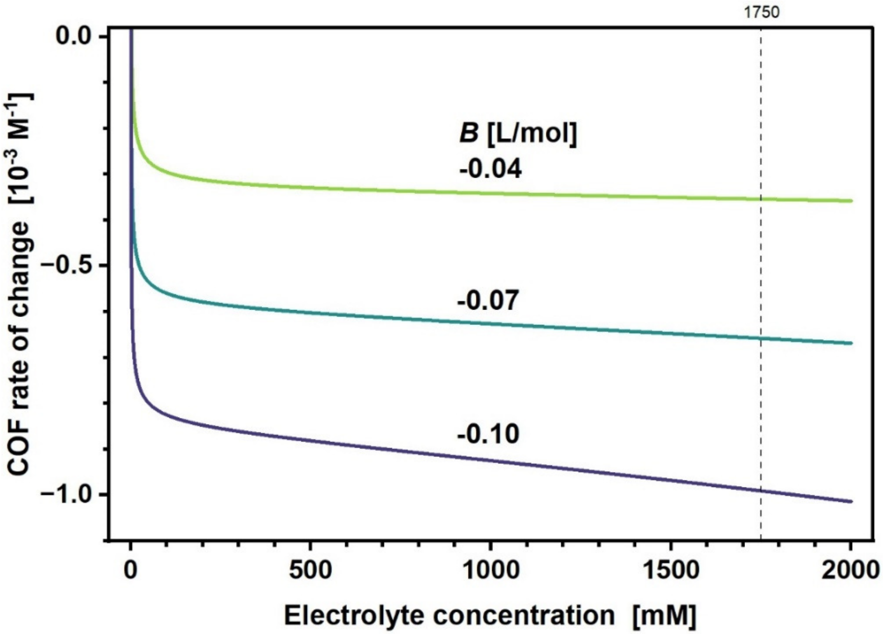

Figure S9. Rates $d\mu/dc$ of calculated COF vs. concentration at the interface (Fig. 2d), for the cases $B =$ -0.04, -0.07 and -0.10 L mol^{-1}. The vertical dashed line marks the nominal electrolyte concentration 25 mM rescaled with the approximate enhancement factor of $f = c_i/c \sim 70$ for the interphasial ion concentration.

S10. Water contact angle of the polycrystalline gold surface

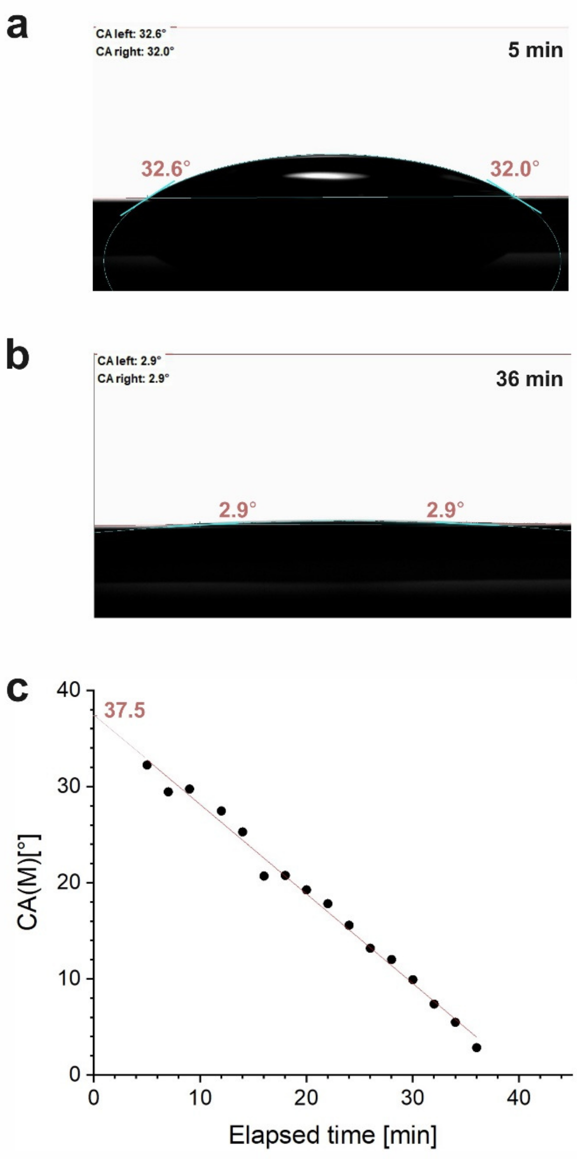

Figure S10. Water contact angle (WCA) analysis. **a** Side view of a sessile drop, imaged ~5 min after removal of the polycrystalline gold film from the plasma cleaner vacuum chamber. **b** Receding drop, imaged after ~36 min. **c** Plot of the mean WCA over the elapsed time.

S11. Hydration layer effects in a tip-sample configuration as described by Feibelman's model

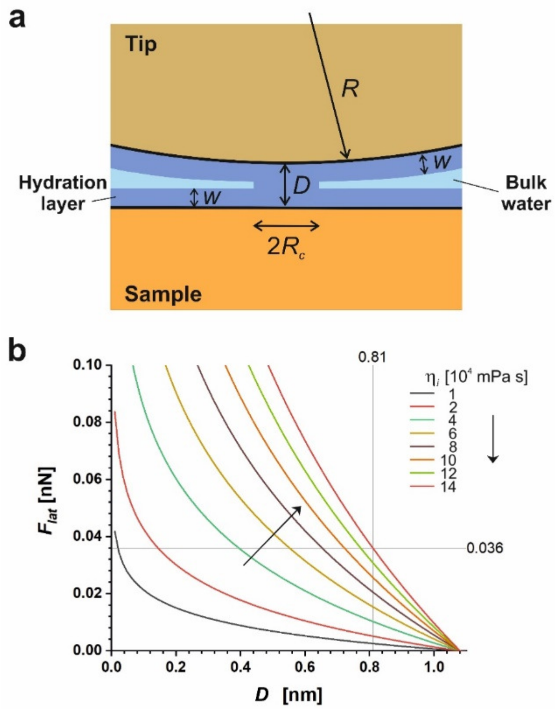

Figure S11. Effect of hydration layers on the lateral force acting on a tip moving across a flat sample surface. **a** Schematic of the tip-sample configuration with hydration layer thickness w and tip-sample distance D. **b** Calculated curves of the lateral force, F_{lat}, as a function of D and the interphasial viscosity η_i, for the case of $w =$ 0.54 nm (corresponding to 2 hydration layers each). In the case of $D =$ 0.81 nm (corresponding to 3 hydration layers), an F_{lat} value of 0.036 nN occurs if $\eta_i = 14 \cdot 10^4$ mPa s. The assumed tip apex radius was R = 79 nm, in line with the value from Fig. S3. The schematic in **a** has been adapted from Fig. 2 in Ref. [17], "Lubrication Theory of Drag on a Probe in Water, near a Hydrophilic Surface", *Langmuir* 22 (2006) 2137, Copyright 2006, American Chemical Society.

Feibelman's model [17] describes the drag force on a parabolic-cylindrical tip moving, in water, parallel to a flat sample surface. Both surfaces are assumed to be covered by a hydration layer of thickness w. At a close distance D, both hydration layers will overlap within a central region of radius R_c. The lateral force acting on the scanning tip is given by

$$F_{lat} = -2\pi\,\eta_i\,R\,v_{lat}\,\Phi\left(D/2w\right) , \qquad \text{(S3)}$$

where η_i, R and v_{lat} denote the interphasial viscosity, tip apex radius and the scan speed, respectively. In the regime $r = D/2w < 1$, the function $\Phi(r)$ can be approximated by $-\ln(r)$ [17].

S12. Interphasial viscosity profiles from molecular dynamics simulation

S12.1 Simulation and system details

Force-field molecular dynamics (MD) simulations similar to those detailed in Ref. [18] were carried out in GPU-enabled single-precision GROMACS 2023.3 [19], [20], using the leap-frog integrator [21] with a time step of 2 fs and a duration of 200 ns. The simulation box is 8.946 $\times$ 6.887 $\times$ 40 nm in size with periodic boundaries along *x*, *y*, and *z*. The velocity-rescaling (CSVR) thermostat is applied to all atoms with a target temperature of 300 K [22]. The Lennard-Jones forces are modelled with force-switching between 1.9 and 2 nm.

Electrostatic forces are modelled using particle-mesh Ewald beyond a real-space cutoff of 2 nm. The simulated system is a decanol-SAM surface with an adsorbed slab of NaCl, KCl, or CsCl electrolyte solution. The SAMs consist of 288 close-packed decanol molecules with a nearest-neighbour distance of 4.97 Å [23–25], which are restrained to fixed points in space by a harmonic potential applied to the bottom carbon of each decanol molecule with spring constant k = 25000 kJ/(mol nm^2). The OH-group partial charges of the decanol molecules are reduced by 10%, giving a neat-water contact angle for the surface of 33° [18]. The liquid consists of 8192 water molecules and the necessary number of anion-cation pairs to achieve the target concentration.

For the simulated electrolytes, concentrations are reported as molalities, i.e. moles of solute per kg of solvent (mol kg^{-1}). In contrast, the experimental concentrations are reported as molarities, i.e. moles of solute per L of solution (mol L^{-1}, denoted as M). For the 500 mmol kg^{-1} solutions, the corresponding molarities are 499.0, 496.8 and 497.4 mM for NaCl, KCl and CsCl, respectively, and for the 5 mol kg^{-1} solutions 4.612, 4.450 and 4.327 M, respectively.

Above the liquid is vacuum in which a vapor phase may form. A quadratic potential a short distance above the liquid-vapor interface acts to reflect any molecules of the vapour back downwards [18]. SAM molecules are modelled using the OPLS All-Atom (OPLS-AA) force field [26–28]. Water is modelled using the SPC/E water model [29]. Solvated ions are modelled using force fields optimized for electrolytes for a wide range of concentrations [30], [31].

A total driving force of 800 kJ/(mol nm^2), which corresponds to a stress of 21.563 MPa, is applied in the *x*-direction to the liquid center of mass, i.e., a force is applied to each atom and ion that is proportional to its mass. In the bulk, where the liquid density is constant, this gives a constant force density, and a quadratic flow profile with the vertex of the parabola at the liquid-vapour interface [18].

Figure S12.I shows the resulting liquid density, velocity, and stress profiles for 500 mmol kg^{-1} and 5 mol kg^{-1} NaCl, KCl and CsCl systems, decomposed into profiles for solvent (water), anion, cation, and total.

The profiles are plotted over $z - z_{GDS}$, where z_{GDS} is the Gibbs dividing surface (GDS) position of the electrolyte. The velocity and stress are the components along the *x*-axis.

S12.2 Viscosity profile extraction via Stokes equation

The extraction of viscosity profiles from driven flow simulations follows the procedure detailed in Ref. [18]. For a system that is translationally invariant in the $x\ y$ – plane, where flow is laminar and in a steady state, the flow is governed by the Stokes equation:

$$f(z) = -\partial_z \eta(z) \partial_z u(z) \quad , \tag{S4}$$

Where u is the liquid velocity, f is the external force density exerted on the liquid, and η is the shear viscosity [18]. For a system consisting of a planar surface in the $x\ y$-plane with an adsorbed liquid slab (where z_0 denotes a position along z below the liquid phase), integration of Eq. (S4) from z_0 to an arbitrary z gives

$$\int_{z_0}^{z} dz' f(z') = -\int_{z_0}^{z} dz' \partial_{z'} \eta(z') u'(z') = -\eta(z) u'(z) \quad , \tag{S5}$$

where the term $\eta(z_0) u'(z_0)$ vanishes because the liquid density is identically zero there, thus giving $\eta(z_0) = 0$, and while $u'(z_0)$ is ill-defined, u' should not diverge in the low-density limit or as the solid surface is approached. Rearranging this equation gives an expression for the viscosity profile

$$\eta(z) = -\frac{1}{u'(z)} \int_{z_0}^{z} dz' f(z') \quad , \tag{S6}$$

which can be calculated numerically from driven-flow simulation data [18]. Here, $f(z)$ consists of the sum of the local applied pulling force density $f_a(z)$ and surface-friction force density $f_f(z)$. Furthermore, $u'(z)$ is the local liquid shear, which can be obtained by taking a numerical derivative of the velocity profile. A similar approach to Eq. (S6) is taken in Refs. [32] and [33], but assuming $F(z) \propto \rho(z)$, where $\rho(z)$ is the liquid density profile, which – crucially – does not account for the surface-liquid friction force.

If Eq. (S6) is implemented correctly, and the force $f(z)$ and velocity $u(z)$ are extracted to a sufficiently high degree of accuracy, the viscosity profile $\eta(z)$ should be found to plateau at a constant

value η_b in the bulk liquid far from the interface. Once η_b is known, Eq. (S4) may be integrated with the alternative boundaries

$$\begin{aligned}&\int_z^{z_b} dz' \, f(z') = -\int_z^{z_b} dz' \partial_{z'} \eta(z') \, u'(z') = \eta(z) u'(z) - \eta_b u'(z_b) \\ &\Rightarrow \eta(z) = \frac{1}{u'(z)} \left(\eta_b u'(z_b) + \int_z^{z_b} dz' \, f(z') \right)\end{aligned} \quad . \qquad \text{(S7)}$$

Equation (S7) is useful for calculating the effective viscosity profile $\eta_{eff}(z)$, where position dependent surface-liquid forces $f_f(z)$ are ignored, with their effects instead being captured in local variations of the (effective) liquid viscosity:

$$\eta_{eff}(z) = \frac{1}{u'(z)} \left(\eta_b u'(z_b) + \int_z^{z_b} dz' \, f_a(z') \right) \quad . \qquad \text{(S8)}$$

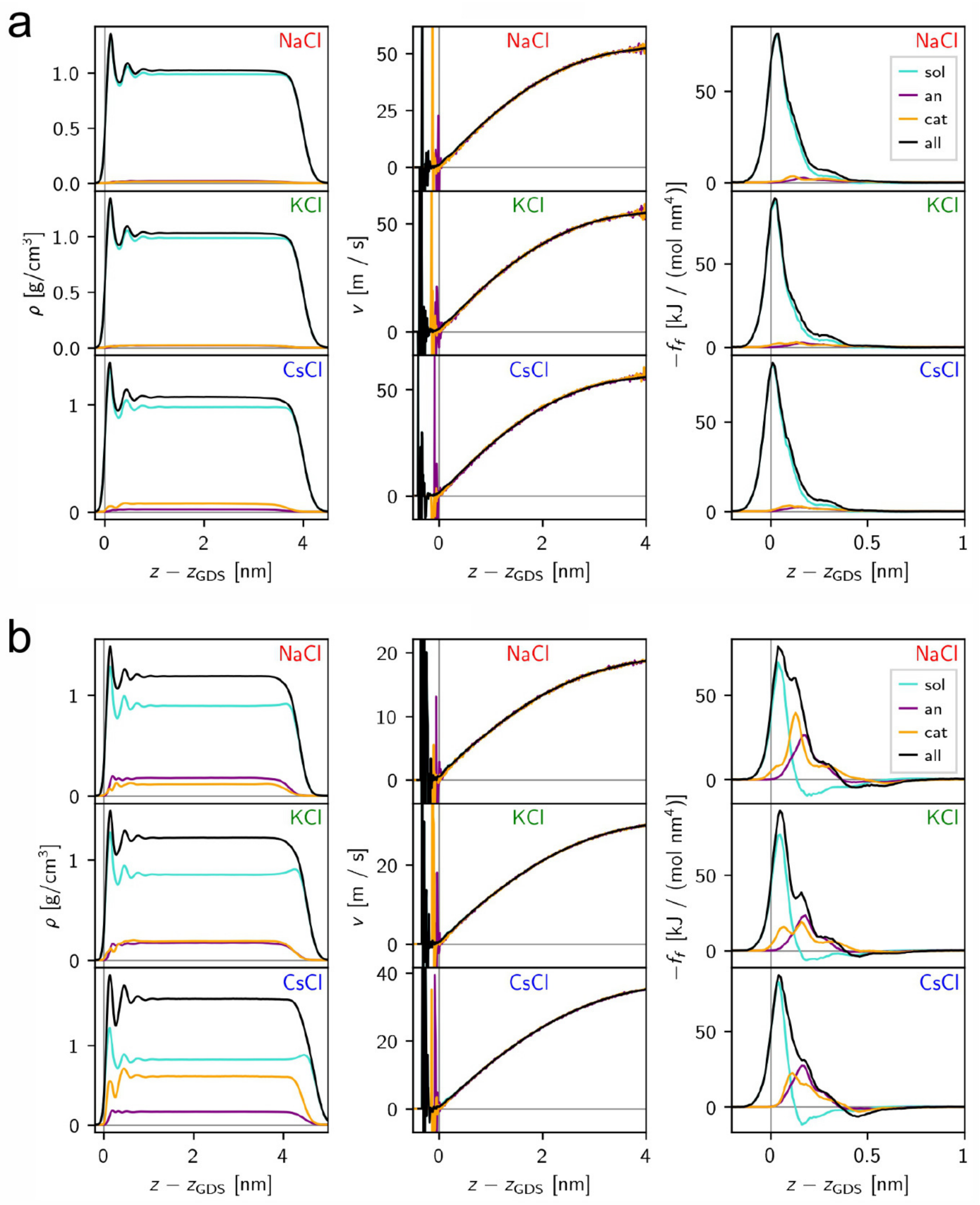

Figure S12.I. Density, velocity and stress profiles from center-of-mass pulling simulations of **a** 500-mmol kg^{-1} and **b** 5-mol kg^{-1} electrolytes on decanol SAMs. In each respective part, the top row is NaCl, the middle KCl and the bottom CsCl. In each plot, the data is decomposed into values for solvent (water), anion, cation and total.

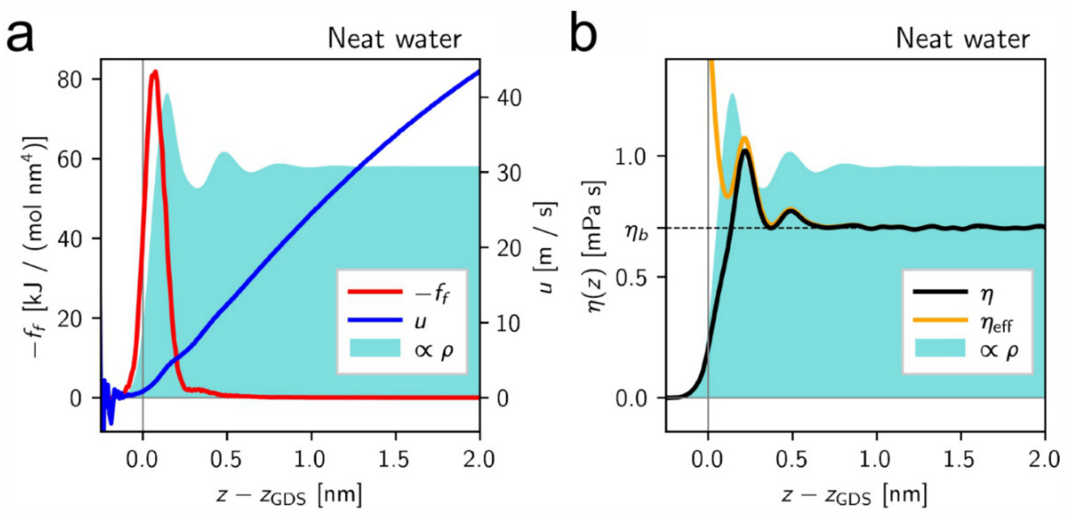

Figure S12.II. Profiles along the surface normal z, extracted from a center-of-mass pulling simulation of neat water on the decanol SAM. The liquid water density is shown as a shaded area (in *cyan*) as a positional reference, scaled by an arbitrary factor. **a** Friction stress $f_f(z)$ and velocity $u(z)$ profiles. **b** Viscosity $\eta(z)$ and effective viscosity $\eta_{eff}(z)$, as calculated via Eqs. (S6) and (S8) respectively.

S12.3 Viscosity profiles for SAM/electrolyte systems

Figure S12.II shows, for the neat-water / SAM system, the raw friction force $f_f(z)$ and velocity $u(z)$ profiles (as in Fig. S12.I) and the resulting viscosity, $\eta(z)$, and effective viscosity, $\eta_{eff}(z)$, profiles (as calculated via Eqs. (S6) and (S8), respectively).

Figure S12.III shows viscosity and effective viscosity profiles for all electrolyte/SAM systems, calculated from the data presented in Fig. S12.I.

Before the viscosity profiles are calculated, the tails of the velocity profiles $u(z)$ are fitted with exponential functions in the region $z - z_{C10} \in [1,2]$Å, where z_{C10} is the mean position of the top carbons of the SAM. The fit data is used in place of the velocity data for $z - z_{C10} \leq 2$ Å. The resulting velocity profile is then smoothed via convolution with a Gaussian kernel with standard deviation $\sigma_{sm} = 0.4$ Å. Only then are the viscosity $\eta(z)$ and effective viscosity $\eta_{eff}(z)$ profiles calculated from Eqs. (S6) and (S8). The dependence on the degree of smoothing, as parameterized by σ_{sm}, is explored in Fig. S12.IV, where $\eta(z)$ is calculated for the 500 mmol kg^{-1} KCl system with various values of σ_{sm}. Here, it is apparent that some smoothing is helpful for the reduction of noise, but the height of the peaks, especially the sharp first peak, nearest the surface, is somewhat reduced by the smoothing.

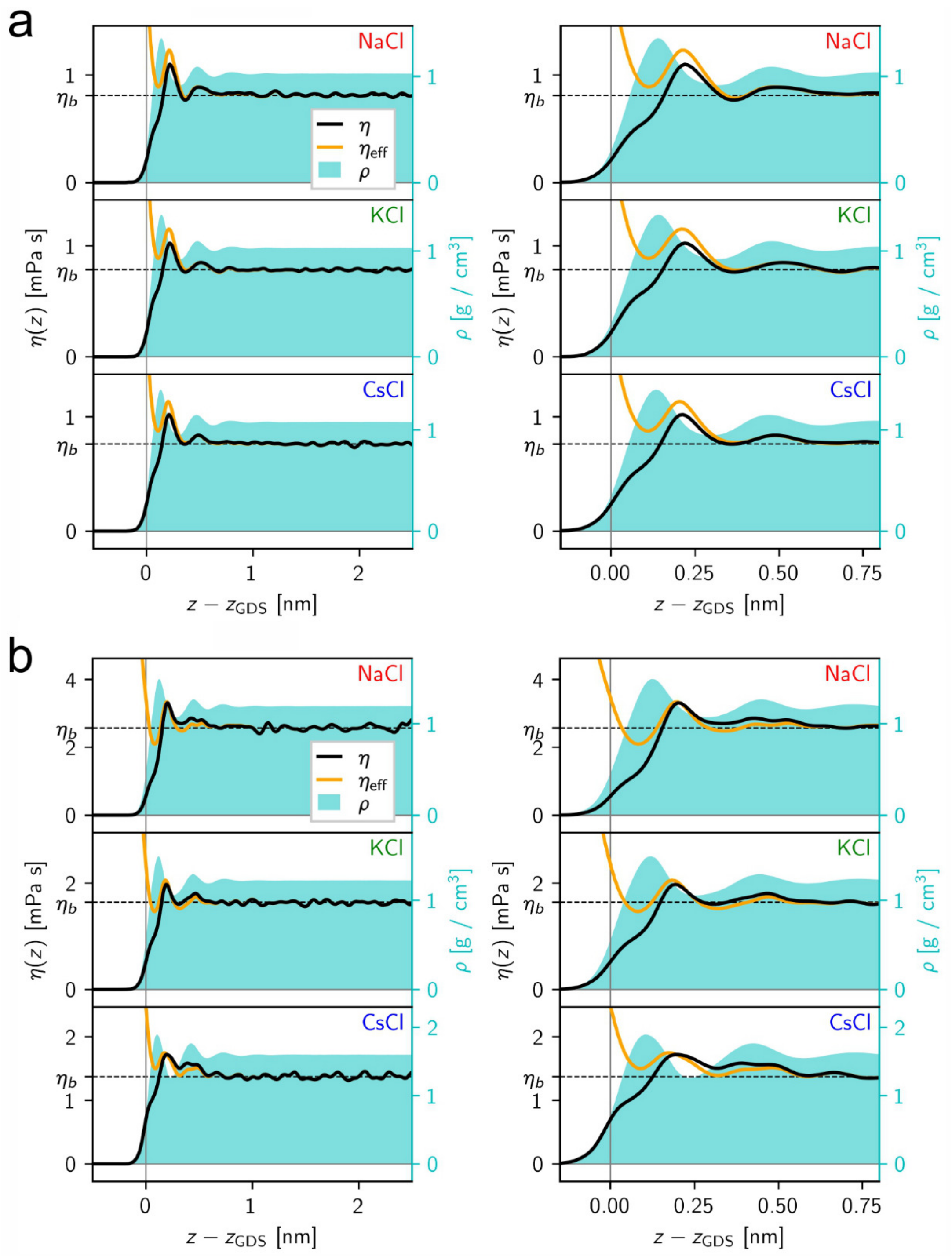

Figure S12.III. Viscosity and effective viscosity profiles, extracted via Eqs. (S6) and (S8), respectively, from center-of-mass pulling simulations of various electrolytes on decanol SAMs. **a** 500- mmol kg^{-1}, **b** 5-mol kg^{-1} electrolyte. In each respective part, profiles for NaCl, KCl and CsCl are shown in the top, middle and bottom rows respectively. While the column on the left shows profile overviews, the column on the right zooms in on the data nearby the interface. The liquid water density is shown as a shaded area (in *cyan*) as a positional reference.

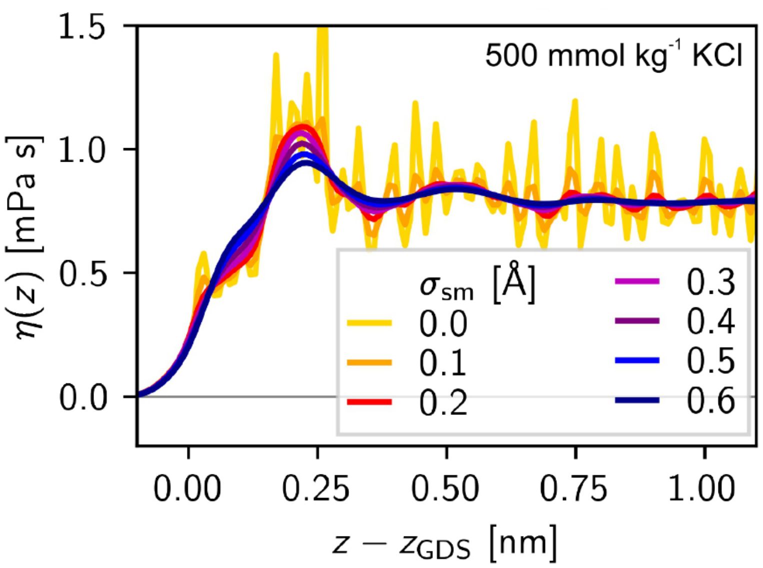

Figure S12.IV. Smoothing of interphasial viscosity profiles from MD simulation, for the case of a 500 mmol kg^{-1} KCl solution. With increasing value of the smoothing parameter, the signal-to-noise ratio (SNR) increases and the characteristic profile features can be seen more clearly. For larger values of 0.5 and 0.6 Å, the height of the 1st peak is reduced. Hence, selection of the value is a trade-off between SNR and peak height.

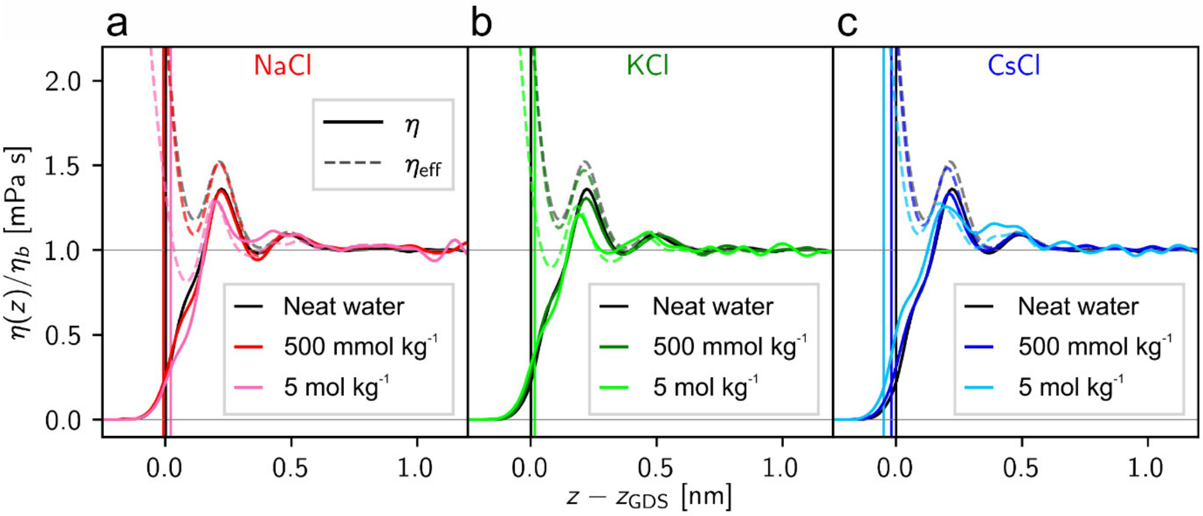

Figure S12.V. Normalized viscosity profiles for **a** NaCl, **b** KCl and **c** CsCl solutions, at 500 mmol kg^{-1} and 5 mol kg^{-1} concentrations. The case of neat water serves as a reference. In each case, a suppression of the 1st peak's height with increasing concentration is observed. The viscosity dividing surfaces are shown as vertical lines.

In Fig. S12.V, the normalized viscosity and effective viscosity profiles are plotted for different concentrations together, for each electrolyte type. In this way, trends in the profile shape as a function of changing concentration can be seen. Most notable is the left and downward shift of the 2nd and

especially 1st peaks with increasing concentration. That is, as the concentration increases, the relative peak size at the surface is reduced. This reflects the fact that the ions increase the viscosity, and there is a deficit of ions at the interface for all electrolyte types (ion deficit distances are approximately between 1.5 and 2.5 Å for all systems).

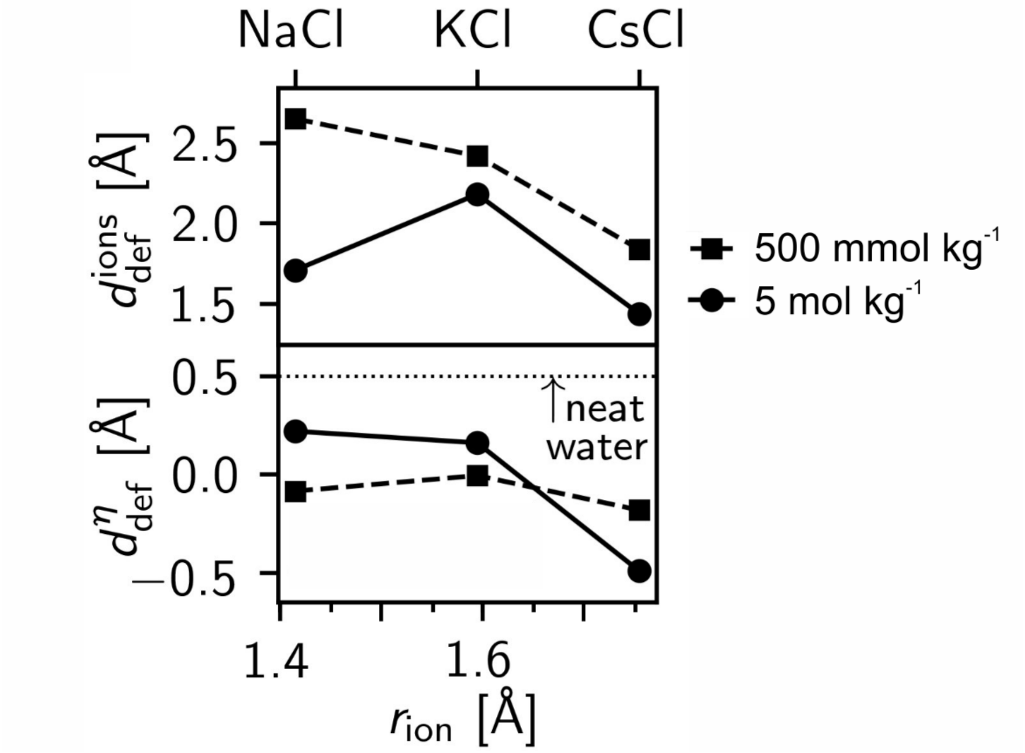

Figure S12.VI. Plots of the ion and viscosity deficit distances. Here, positive values indicate a deficit of ion density and viscosity, respectively, at the interface. The viscosity deficit distance d_{def}^{η} denotes the displacement $z_{GDS} - z_{\eta}$ of the viscosity dividing surfaces (where the liquid viscosity excess vanishes) away from the Gibbs dividing surface (GDS) of the respective liquid.

S13. Jones-Dole *B*–coefficient *versus* ion radius

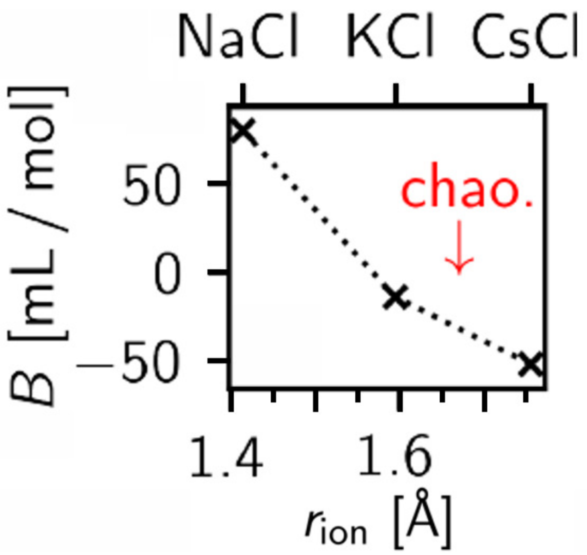

Figure S13. Plot of the Jones-Dole B – coefficient vs. the bare (i.e., non-hydrated) ion radius, r_{ion}, using data reported in Ref. [7].

References

[1] Varenberg M, Etsion I and Halperin G 2003 An improved wedge calibration method for lateral force in atomic force microscopy *Rev. Sci. Instrum.* **74** 3362

[2] Ruan J-A and Bhushan B 1994 Atomic-Scale Friction Measurements Using Friction Force Microscopy: Part I—General Principles and New Measurement Techniques *J. Tribol.* **116** 378–88

[3] Tong Y, Lapointe F, Thämer M, Wolf M and Campen R K 2017 Hydrophobic Water Probed Experimentally at the Gold Electrode/Aqueous Interface *Angew. Chemie Int. Ed.* **56** 4211–4

[4] Bongaerts J H H, Fourtouni K and Stokes J R 2007 Soft-tribology: Lubrication in a compliant PDMS–PDMS contact *Tribol. Int.* **40** 1531–42

[5] Wang Y, Wang Q J, Lin C and Shi F 2006 Development of a Set of Stribeck Curves for Conformal Contacts of Rough Surfaces *Tribol. Trans.* **49** 526–35

[6] Jones G and Dole M 1929 The viscosity of aqueous solutions of strong electrolytes with special reference to barium chloride *J. Am. Chem. Soc.* **51** 2950–64

[7] Jenkins H D B and Marcus Y 1995 Viscosity B-Coefficients of Ions in Solution *Chem. Rev.* **95** 2695–724

[8] dos Santos A P, Diehl A and Levin Y 2010 Surface Tensions, Surface Potentials, and the Hofmeister Series of Electrolyte Solutions *Langmuir* **26** 10778–83

[9] Leppin C, Peschel A, Meyer F S, Langhoff A and Johannsmann D 2021 Kinetics of viscoelasticity in the electric double layer following steps in the electrode potential studied by a fast electrochemical quartz crystal microbalance (EQCM) *Analyst* **146** 2160–71

[10] Grierson D S, Flater E E and Carpick R W 2005 Accounting for the JKR–DMT transition in adhesion and friction measurements with atomic force microscopy *J. Adhes. Sci. Technol.* **19** 291–311

[11] Fréchtt J and Vandrlick T K 2005 Elctrocapillary at Contact: Potntial-Dpndnt Adhsion btwn a Gold Elctrod and a Mica Surfac *Langmuir* **21** 985–91

[12] Iler R K 1979 *The Chemistry of Silica* (New York: Wiley-Interscience)

[13] Batteas J D, Weldon M K and Raghavachari K 2003 Bonding and Interparticle Interactions of Silica Nanoparticles *Nanotribology* ed S M Hsu and Z C Ying (Norwell: Kluwer Academic Publishers) pp 387–98

[14] Wang J and Bard A J 2001 Direct Atomic Force Microscopic Determination of Surface Charge at the Gold/Electrolyte InterfaceThe Inadequacy of Classical GCS Theory in Describing the Double-Layer Charge Distribution *J. Phys. Chem. B* **105** 5217–22

[15] Pezzotti G 2023 Silicon nitride as a biomaterial *J. Ceram. Soc. Japan* **131** 398–428

[16] Dishon M, Zohar O and Sivan U 2009 From Repulsion to Attraction and Back to Repulsion: The Effect of NaCl, KCl, and CsCl on the Force between Silica Surfaces in Aqueous Solution *Langmuir* **25** 2831–6

[17] Feibelman P J 2006 Lubrication Theory of Drag on a Scanning Probe in Structured Water, near a Hydrophilic Surface *Langmuir* **22** 2136–40

[18] Carlson S R and Netz R R 2025 Subnanometer Interfacial Hydrodynamics: Spatially Resolved

Viscosity and Surface Friction *Nano Lett.* **25** 15605–12

[19] Berendsen H J C, van der Spoel D and van Drunen R 1995 GROMACS: A message-passing parallel molecular dynamics implementation *Comput. Phys. Commun.* **91** 43–56

[20] Lindahl E, Hess B and van der Spoel D 2001 GROMACS 3.0: a package for molecular simulation and trajectory analysis *J. Mol. Model.* **7** 306–17

[21] Hockney R W, Goel S P and Eastwood J W 1974 Quiet high-resolution computer models of a plasma *J. Comput. Phys.* **14** 148–58

[22] Bussi G, Donadio D and Parrinello M 2007 Canonical sampling through velocity rescaling *J. Chem. Phys.* **126** 14101

[23] Strong L and Whitesides G M 1988 Structures of self-assembled monolayer films of organosulfur compounds adsorbed on gold single crystals: electron diffraction studies *Langmuir* **4** 546–58

[24] Chidsey C E D and Loiacono D N 1990 Chemical functionality in self-assembled monolayers: structural and electrochemical properties *Langmuir* **6** 682–91

[25] Ulman A, Eilers J E and Tillman N 1989 Packing and molecular orientation of alkanethiol monolayers on gold surfaces *Langmuir* **5** 1147–52

[26] Jorgensen W L, Madura J D and Swenson C J 1984 Optimized intermolecular potential functions for liquid hydrocarbons *J. Am. Chem. Soc.* **106** 6638–46

[27] Kaminski G, Duffy E M, Matsui T and Jorgensen W L 1994 Free Energies of Hydration and Pure Liquid Properties of Hydrocarbons from the OPLS All-Atom Model *J. Phys. Chem.* **98** 13077–82

[28] Jorgensen W L, Maxwell D S and Tirado-Rives J 1996 Development and Testing of the OPLS All-Atom Force Field on Conformational Energetics and Properties of Organic Liquids *J. Am. Chem. Soc.* **118** 11225–36

[29] Berendsen H J C, Grigera J R and Straatsma T P 1987 The missing term in effective pair potentials *J. Phys. Chem.* **91** 6269–71

[30] Loche P, Steinbrunner P, Friedowitz S, Netz R R and Bonthuis D J 2021 Transferable Ion Force Fields in Water from a Simultaneous Optimization of Ion Solvation and Ion–Ion Interaction *J. Phys. Chem. B* **125** 8581–7

[31] Duenas-Herrera M, Bonthuis D J, Loche P, Netz R R and Scalfi L 2024 Force field for halide and alkali ions in water based on single-ion and ion-pair thermodynamic properties for a wide range of concentrations *J. Chem. Phys.* **161** 74506

[32] Todd B D, Evans D J and Daivis P J 1995 Pressure tensor for inhomogeneous fluids *Phys. Rev. E* **52** 1627–38

[33] Akhmatskaya E, Todd B D, Daivis P J, Evans D J, Gubbins K E and Pozhar L A 1997 A study of viscosity inhomogeneity in porous media *J. Chem. Phys.* **106** 4684–95
